# Allele-specific genome editing and correction of disease-associated phenotypes in rats using the CRISPR–Cas platform

**DOI:** 10.1038/ncomms5240

**Published:** 2014-06-26

**Authors:** K. Yoshimi, T. Kaneko, B. Voigt, T. Mashimo

**Affiliations:** 1 Institute of Laboratory Animals, Graduate School of Medicine, Kyoto University, Kyoto 606-8501, Japan

## Abstract

The bacterial CRISPR/Cas system has proven to be an efficient gene-targeting tool in various organisms. Here we employ CRISPR/Cas for accurate and efficient genome editing in rats. The synthetic chimeric guide RNAs (gRNAs) discriminate a single-nucleotide polymorphism (SNP) difference in rat embryonic fibroblasts, allowing allele-specific genome editing of the dominant phenotype in (F344 × DA)F1 hybrid embryos. Interestingly, the targeted allele, initially assessed by the allele-specific gRNA, is repaired by an interallelic gene conversion between homologous chromosomes. Using single-stranded oligodeoxynucleotides, we recover three recessive phenotypes: the albino phenotype by SNP exchange; the non-agouti phenotype by integration of a 19-bp DNA fragment; and the hooded phenotype by eliminating a 7,098-bp insertional DNA fragment, evolutionary-derived from an endogenous retrovirus. Successful *in vivo* application of the CRISPR/Cas system confirms its importance as a genetic engineering tool for creating animal models of human diseases and its potential use in gene therapy.

## Abstract (web summary)

The bacterial CRISPR–Cas system is increasingly used for genome editing in animal models. Here the authors utilize this system to target and edit specific coat colour alleles in rats and demonstrate the potential of this technology for the creation of genetically engineered animal models.

## Graphical abstract

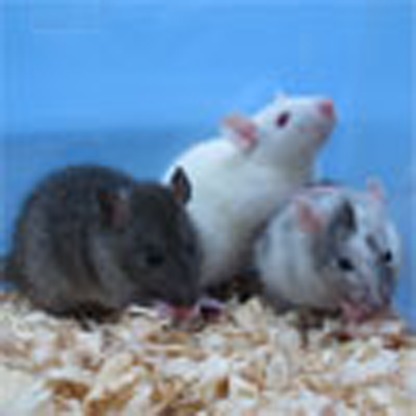

The laboratory rat, *Rattus norvegicus*, is a widely used animal model for studying human diseases, such as hypertension^1^, diabetes^2^, neurological disorders^3^ and for testing the efficacy and toxicity of drugs. Because of its larger body size compared with mice and their physiological properties, which are being shared with humans, the rat is often employed as an animal model in translational research^4^,^5^. Recent progress in the development of genome engineering tools in rats, such as zinc-finger nucleases (ZFNs)^6^,^7^,^8^ and transcription activator-like effector nucleases (TALENs)^9^,^10^, could provide genetically modified animals for gene annotation, as well as for modelling human genetic disorders. These engineered nucleases can recognize long stretches of DNA sequences and introduce DNA double-strand breaks (DSBs), which are generally restored via non-homologous end-joining, a process that introduces small insertions or deletions (indels) at the repair junction, thereby generating knockouts (KOs) at the targeted sequences. Targeted knock-ins (KIs) can also be engineered via homology-directed repair (HDR) by co-injection into fertilized eggs of donor plasmids containing two flanked homology arms together with any of the above mentioned nucleases^6^,^11^,^12^. Although the nuclease-driven production of targeted KOs or KIs is simple and fast, HDR-mediated KIs are less efficiently to obtain.

Over the last decade, the emergent technology of next-generation sequencing, which has powered genome-wide association studies, has successfully identified numerous common single-nucleotide polymorphisms (SNPs) associated with important human diseases^13^,^14^,^15^. Many structural sequence variations, such as small-scale indels, copy-number variations and large chromosomal rearrangements have been identified^16^,^17^. To test these particular structural variants in model animals, accurate genome editing is required to produce equivalent mutations to human variants rather than producing only simple KO models where entire coding genes are rendered non-functional.

The bacterial CRISPR/Cas system has been shown to be an efficient gene-targeting technology in mammalian cells^18^,^19^,^20^ and many organisms^21^,^22^,^23^,^24^,^25^, including mice^26^,^27^ and rats^12^,^28^,^29^. The system consists of clustered regularly interspaced short palindromic repeats (CRISPRs) that produce RNA components and the CRISPR-associated (Cas) nuclease protein^30^,^31^,^32^. The CRISPR RNAs (crRNAs), which contain a short stretch of homology to a specific target DNA, act as guides to direct Cas nucleases to introduce DSBs at the targeted DNA sequences. A synthetic chimeric guide RNA (gRNA), consisting of a fusion between crRNA and trans-activating crRNA, has been shown to direct Cas9 cleavage of target DNAs that are complementary to the crRNA. In addition to the rapid creation of synthetic gRNAs, a significant advantage of the CRISPR/Cas system is its ability to target several genes simultaneously with multiple gRNAs (multiplex gene editing). Another advantage of the CRISPR/Cas system has been described from findings in mice^26^,^27^,^33^. These results suggest that co-injected single-stranded oligodeoxynucleotides (ssODNs) as donor templates preferentially support the activation of HDR relative to the non-homologous end-joining pathway.

In this study, we construct CRISPR/Cas architectures in rats, and show allele-specific KOs of the dominant allele (Supplementary Fig. 1). We also correct the three recessive coat-colour-associated phenotypes that are responsible for the appearance of all ‘albino-white’ laboratory rats using a KI approach that uses ssODN donor templates. These results demonstrate the flexible *in vivo* genome-editing capability of the CRISPR/Cas system and its usability for the creation of genetically engineered animal models of human diseases in rats.

## Results

### Accurate and efficient genome editing in rats

**Figure 1:**
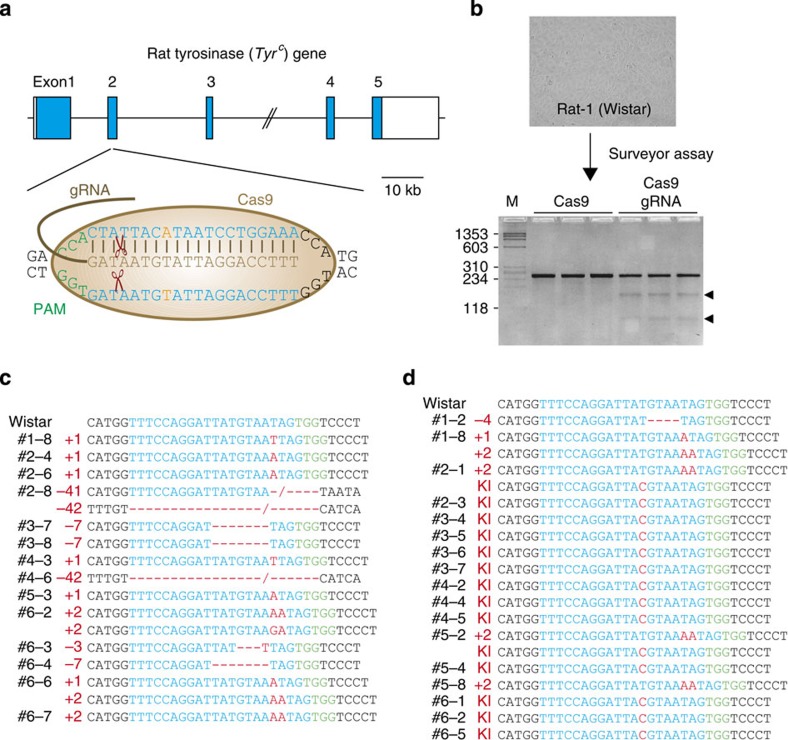
NHEJ-mediated KO and HDR-mediated KI in Wistar rats using the CRISPR/Cas system. (**a**) Schematic representation of the rat tyrosinase (*Tyr*) gene. The magnified view illustrates the gRNA binding sites (blue) and the PAM sequences (green). Wistar albino rats carry a G896A SNP mutation (orange) in exon 2 of the *Tyr* gene. (**b**) Plasmids expressing gRNA and codon-optimized Cas9 were transfected into Wistar-derived Rat-1 fibroblasts. The Surveyor (Cel-I) nuclease assay on exon 2 of *Tyr* showed targeted cleavage of the digested PCR products (indicated by arrowheads). M: DNA marker phiX174-HaeIII digest. Cas9: Cas9-transfected Rat-1. Cas9 gRNA: Cas9 and gRNA plasmid -transfected Rat-1. (**c**) Microinjection of gRNA and Cas9 mRNA into fertilized Wistar rat eggs. Sequence analysis of PCR products amplified from the genomic DNA of two-cell embryos showed a wide variety of indel mutations mediated by NHEJ at the targeted *Tyr* exon 2 (see also Table 1). (**d**) Co-injection of gRNA, Cas9 mRNA, and ssODN into fertilized Wistar rat eggs. Sequence analysis showed indel mutations at the targeted *Tyr* exon 2 as well as the precise SNP exchange mediated by HDR that resulted in KI alleles (see also Table 1).

To test the feasibility of genome editing using the CRISPR/Cas system in rats, we first designed gRNA-targeting of the rat coat colour gene, tyrosinase (*Tyr*) (Fig. 1a). To decrease the possibility of off-target (OT) effects, we used software tools that can predict unique and suitable target sites throughout the rat genome (crispr.mit.edu)^34^. Then, we transfected plasmids expressing the engineered gRNA and codon-optimized Cas9 into the cultured rat fibroblast-like cell line (Rat-1) derived from Wistar rats. Compared with the negative control, which comprised only Cas9-transfected cells, the cells transfected with Cas9 and gRNA showed targeted cleavage of the PCR products determined by the Surveyor (Cel-I) nuclease assay (Fig. 1b). Sequence analysis of the targeted *Tyr* locus also showed a wide variety of indel mutations with a targeted cleavage efficiency of 31.6% (Supplementary Fig. 2).

Next, we investigated the capability of CRISPR/Cas to direct targeted cleavage in rat embryos, by microinjection of 50 ng μl^−1^ gRNA and 100 ng μl^−1^ Cas9 messenger RNA (mRNA) into male pronuclei of fertilized Wistar rat eggs (Table 1). After 16 h, 41 of the 90 Cas9/gRNA-injected embryos differentiated normally into two cells (45.6%). Of 34 PCR-amplified two-cell embryos, 14 (41.2%) showed a variety of indel mutations mediated by CRISPR/Cas at the targeted *Tyr* locus (Fig. 1c). Furthermore, 10 two-cell Cas9/gRNA-injected embryos were transferred into a pseudopregnant foster mother, and three of these embryos were carried to term. Sequence analyses of their tail DNA revealed that all these pups carried indel mutations that were heterozygous or mosaic at the *Tyr* locus (Supplementary Fig. 3). Crossing these founders with Wistar rats demonstrated that all of the CRISPR/Cas-mediated mutations were faithfully transmitted to the next generation (Supplementary Table 1). In addition, neither insertions nor deletions were observed at any of the seven most likely calculated OT sites identified across the whole rat genome with a similarity to the targeted site of 3- to 5-bp mismatches from the 20-bp binding sequences and protospacer adjacent motif sequences (Supplementary Table 2).

In mice^26^,^27^, co-injection of gRNAs, Cas9 mRNA and ssODNs has been reported to allow for precise HDR-mediated genome editing. To test this, the pronuclei of Wistar rat eggs were microinjected with gRNA, Cas9 mRNA and 50 ng μl^−1^ ssODN (Table 1). Of the 38 two-cell developed and PCR-amplified embryos, 5 (13.2%) showed various indel mutations at the targeted loci. Surprisingly, 14 (36.8%) showed a precise SNP exchange mediated by ssODN-HDR, among which two were biallelic with indel mutations (#2-1 and #5-2) and three carried homozygous KI alleles (#2-3, #5-4 and #6-2) at the *Tyr* locus (Fig. 1d).

### Allele-specific genome editing for a dominant phenotype

**Figure 2:**
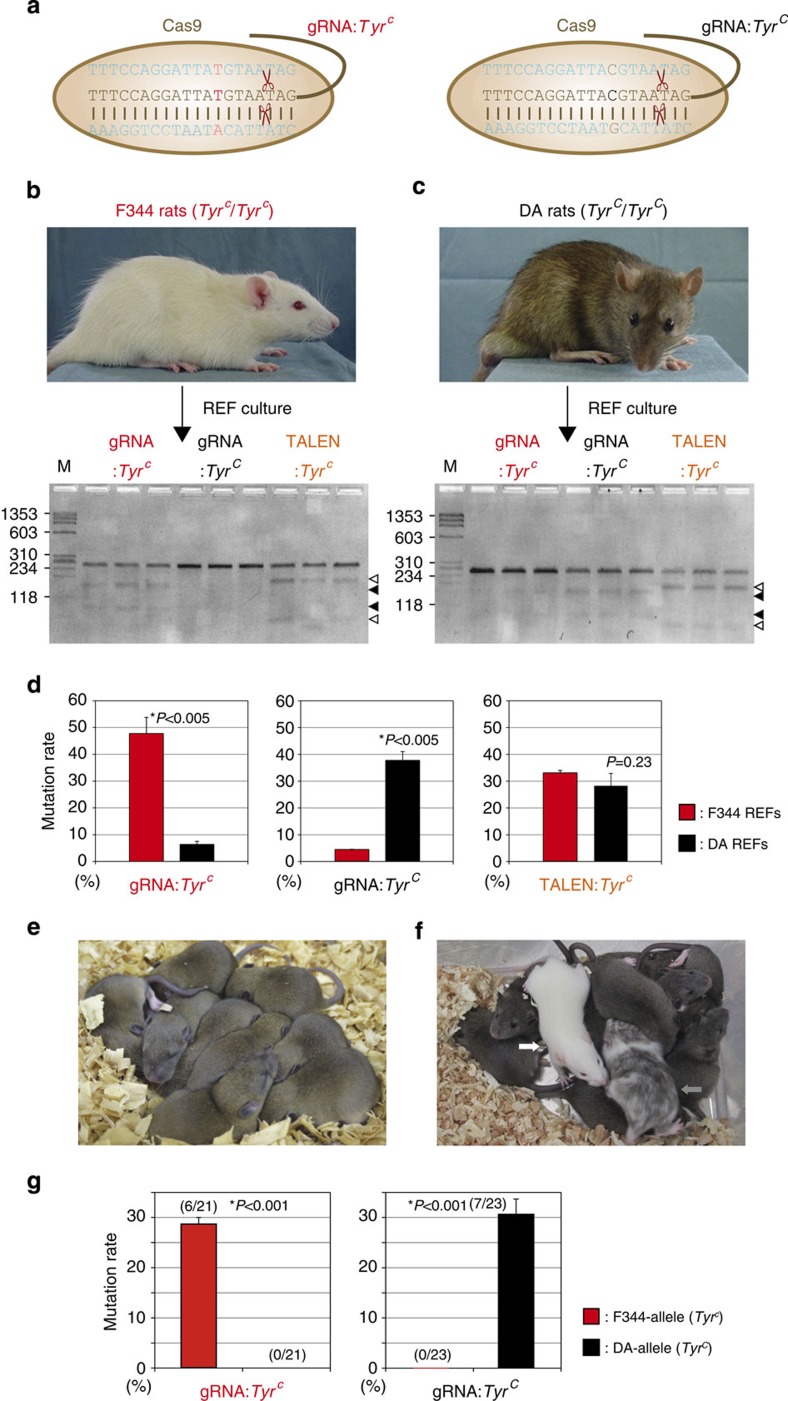
Allele-specific genome editing in F1 rats by the CRISPR/Cas system. (**a**) Schematic representation of gRNA:*Tyr*^*c*^ targeting of the mutant allele (*Tyr*^*c*^) of albino F344 rats and gRNA:*Tyr*^*C*^ targeting of the wild-type allele (*Tyr*^*C*^) of agouti DA rats. (**b**) Plasmids expressing Cas9 and allele-specific gRNA transfected into F344-derived rat REFs. Cleavage activity by the Surveyor assay was detected with gRNA:*Tyr*^*c*^ and with TALENs targeting *Tyr*^*c*^ (as a positive control), but not with gRNA:*Tyr*^*C*^. M: DNA marker phiX174-HaeIII digest. (**c**) In DA-derived REFs, cleavage activity was detected with gRNA:*Tyr*^*C*^ and with TALENs targeting of *Tyr*^*c*^, but not with gRNA:*Tyr*^*c*^. (**d**) Sequence analysis of the colonies picked from subcloned PCR products from the cultured REFs (**b**,**c**). The allele-specific gRNA, and gRNA:*Tyr*^*c*^ and gRNA:*Tyr*^*C*^ showed allele-specific cleavage activity in F344 and DA REFs, respectively, but the TALENs did not act in an allele-specific manner. Data represent the mean±s.d., *n*=3. *P<0.005 by Student’s *t*-test. (**e**) Picture of gRNA:*Tyr*^*c*^-injected (F344 × DA)F1 and (F344 × DA)F1 rats showing the *Agouti* coat-colour. (**f**) Some of the gRNA:*Tyr*^*C*^-injected F1 rats had albino coloured coats (white arrow) or mosaic coloured coats (grey arrow). (**g**) Sequence analysis for each of the gRNA-injected F1 hybrid rats. gRNA:*Tyr*^*c*^ modified only the F344-allele (*Tyr*^*c*^), while gRNA:*Tyr*^*C*^ modified only the DA-allele (*Tyr*^*C*^) in the F1 hybrid rats. Data represent the mean±s.d., *n*=3. *P<0.001 by Student’s *t*-test.

The high efficiency of the CRISPR/Cas system-mediated genome editing in rats prompted us to modify observable phenotypic traits, or to replace disease-causing mutations as therapeutic models of human diseases. In humans, mutations in the *TYR* gene with impaired TYR protein levels lead to oculocutaneous albinism type 1 (OCA1), characterized by hypopigmentation of the skin and hair and distinctive ocular changes^35^. Albino rats carry a single SNP mutation 896G>A in exon 2 of the *Tyr* gene resulting in an Arg299His missense mutation, which was also reported in human oculocutaneous albinism type 1A with lack of pigmentation^36^,^37^. To test disease-specific genome editing using the CRISPR/Cas system, we designed two gRNAs: gRNA:*Tyr*^*c*^ for the mutant allele (*Tyr*^*c*^) of albino F344 rats, and gRNA:*Tyr*^*C*^ targeting the wild-type allele (*Tyr*^*C*^) of *agouti* DA rats (Fig. 2a). We also used TALENs for targeting the albino *Tyr*^*c*^ allele as a control^9^. When we transfected plasmids expressing the Cas9 and the allele-specific gRNA into rat embryonic fibroblasts (REFs) derived from the albino F344 rats, cleavage activity was detected by the Surveyor assay with gRNA:*Tyr*^*c*^, but not with gRNA:*Tyr*^*C*^ (Fig. 2b). In contrast, in REFs derived from DA rats, gRNA:*Tyr*^*C*^ exhibited cleavage activity, while gRNA:*Tyr*^*c*^ did not (Fig. 2c). Our previously designed TALENs, which were designed to target only the albino *Tyr*^*c*^ allele^9^, could in fact not distinguish between the *Tyr* albino/non-albino allele and showed similar cleavage activity in both F344 REFs and DA REFs (Fig. 2b,c). Sequence analysis of the PCR products also confirmed that each gRNA showed significant allele specificity in each of the F344 and DA REF cell types (Fig. 2d; Supplementary Figs 4 and 5), whereas the TALENs did not (Fig. 2d; Supplementary Fig. 6).

To investigate whether the allele-specific genome editing used herein was feasible in embryos, we injected each gRNA with Cas9 mRNA into the fertilized eggs of (F344 × DA)F1 hybrids (Table 2). Transferring the injected embryos into pseudopregnant Wistar females resulted in 21 pups born from gRNA:*Tyr*^*c*^-injected embryos and 23 pups born from gRNA:*Tyr*^*C*^-injected embryos. All of the pups injected with gRNA:*Tyr*^*c*^ showed Agouti coat colour (Fig. 2e), while seven out of 23 pups (30.4%) injected with gRNA:*Tyr*^*C*^ showed albino- or mosaic-coloured coats (Fig. 2f). Sequence analysis revealed that gRNA:*Tyr*^*c*^ only induced indel mutations in the F344 *Tyr*^*c*^ allele (6 mutations/21 alleles), while gRNA:*Tyr*^*C*^ only did so in the DA *Tyr*^*C*^ allele (7/23) in F1 hybrid embryos (Fig. 2g; Supplementary Figs 7 and 8). These findings indicate that the CRISPR/Cas system can discriminate single-base pair differences and target allele-specific DNA variants in embryos. Another interesting finding of this allele-specific targeting is that two of the seven (28.6%) albino coat colour F1 rats injected with gRNA:*Tyr*^*C*^ carried homologous F344 *Tyr*^*c*^alleles (Supplementary Fig. 8), suggesting that an interallelic gene conversion event occurred between the CRISPR/Cas-mediated DA allele and the non-modified F344 allele, in which the original non-albino DA allele was allele-specifically cut and the untouched albino F344 allele served as a repair template.

### Recovery of recessive disease-associated mutations

**Figure 3:**
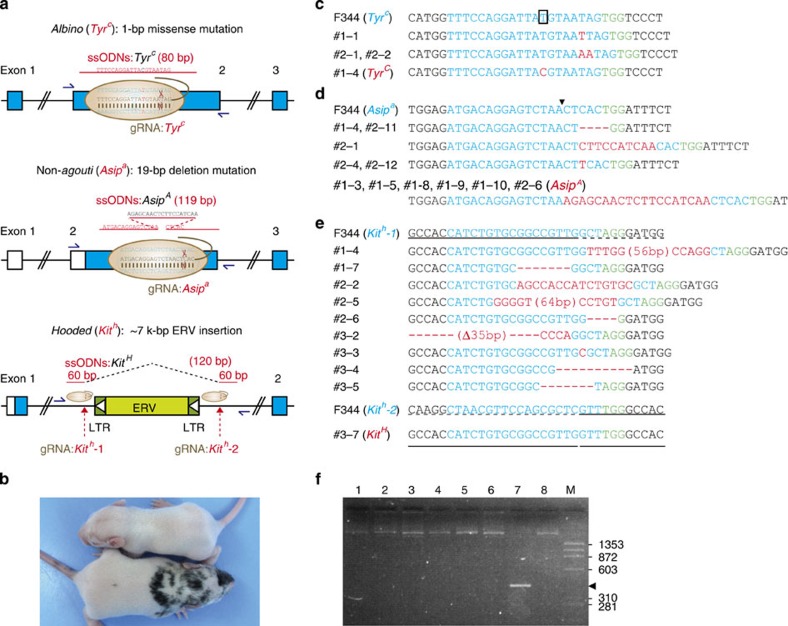
Recovery of three distinct coat-colour mutations by CRISPR/Cas. (**a**) Schematic illustration of three coat-colour mutations in rats. *albino* (*Tyr*^*c*^): SNP missense mutation in the *Tyr* gene. *non-agouti* (*Asip*^*a*^): 19-bp deletion in exon 2 of the Agouti signalling protein (*Asip*) gene. *hooded* (*Kit*^*h*^): integration of an 7,098-bp endogenous retrovirus (ERV) element within the first intron of the *Kit* gene. (**b**) Coat-colour phenotypes (*C, a, h*) recovered from *albino* by injecting gRNA:*Tyr*^*c*^, Cas9 mRNA, and ssODN of the *Tyr*^*C*^ allele into F344 rat embryos (*c, a, h*). (**c**) Sequence analysis of the targeted *Tyr* exon 2 in the injected F344 rats. Cas9 and gRNA with ssODN mediated the introduction of several indel mutations, and the precise HDR-mediated SNP exchange of *Tyr*^*C*^. (**d**) Recovery of the *non-agouti* phenotype by injecting gRNA:*Asip*^*a*^, Cas9 mRNA, and ssODN of the *Asip*^*A*^ allele into F344 rat embryos. Cas9 and gRNA with ssODN mediated the introduction of several indel mutations, and the precise short DNA fragment integration of the *Asip*^*A*^ *gene*. (**e**) Recovery of the *hooded* mutation by injecting gRNA:*Kit*^*h*^*-1*, gRNA:*Kit*^*h*^*-2*, and ssODN of *the Kit*^*H*^ allele into F344 rat embryos. Cas9 and two gRNAs with ssODN mediated the introduction of indel mutations at the targeted *Kit*^*h*^*-1* locus, and the precise large deletion between the two cutting edges of *Kit*^*H*^. (**f**) PCR analysis of the injected F344 rats using primers designed against each outer side of the two LTR sequences. M: DNA marker phiX174-HaeIII digest.

To test whether the CRISPR/Cas platform can be used to recover recessive disease-associated phenotypes, we targeted three different types of mutations associated with representative coat-colour phenotypes in rats: albino (*c*) a SNP missense mutation in the *Tyr* gene^30^, non-agouti (*a*) a 19-bp deletion in exon 2 of the Agouti-signalling protein (*Asip*) gene^31^, and hooded (*h*) an integrated 7,098-bp endogenous retroviral element (ERV) within the first intron of the *Kit* gene^32^ (Fig. 3a). F344 rats carry all three of these mutations (*c*, *a*, *h*), while DA rats carry wild-type alleles (*C*, *A*, *H*). First, to recover the albino phenotype, we co-injected a mix of gRNA:*Tyr*^*c*^, Cas9 mRNA and an 80-bp ssODN of the *Tyr*^*C*^ allele into F344 rat embryos (Table 3). Of the 13 pups delivered, one pup (7.7%) showed a recovery of the coat-colour for albino with non-agouti, hooded phenotype (*C*, *a*, *h*) (Fig. 3b). Sequence analysis at the targeted *Tyr* locus revealed indel mutations in three pups (23.1%), and the precise SNP exchange in one pup (7.7%) (Fig. 3c; Table 3).

Next, by injecting Cas9-gRNA:*Asip*^*a*^ with ssODN:*Asip*^*A*^ into F344 embryos, we attempted targeted integration of a 19-bp nucleotide fragment at the *Asip*^*a*^ locus to recover the non-agouti phenotype (Fig. 3a). Sequence analysis revealed that 5 of the 33 pups injected carried indel mutations (15.2%) at the targeted *Asip*^*a*^ locus while 6 pups carried the precisely repaired *Asip*^*A*^ allele KI (18.2%) (Fig. 3d; Table 3). To recover the hooded phenotype by eliminating the large ≈7-kb fragment including an ERV and two long terminal repeat sequences at either ends of the ERV, we used two gRNAs:*Kit*^*h*^*-1* and:*Kit*^*h*^*-2* modules designed against the outside sequences of the two long terminal repeats, and ssODN:*Kit*^*H*^consisting of two times 60-bp each located at the outer sides of the Cas9-cutting edges (Fig. 3a). Of the 25 pups that were injected, 9 (36.0%) showed indel mutations at the *Kit*^*h*^*-1* locus, but no pups carried any mutations at the *Kit*^*h*^*-2* locus (Fig. 3e; Table 3). However, DNA from one pup produced a positive PCR band (405 bp) amplified by a primer set designed for the outer sides adjacent to the two gRNA-targeting sequences (Fig. 3f). Sequence analysis confirmed that the two cutting edges were accurately joined, thereby comprising the final 120-bp ssODN:*Kit*^*H*^ module (Fig. 3e). Finally, crossing the *Asip*^*A*^ founder (*c*, *A*, *h*) and the *Kit*^*H*^ founder (*c*, *a*, *H*) mediated by CRISPR/Cas with black-hooded PVG/Seac rats (*C*, *a*, *h*) resulted in the recovery of Agouti-hooded (*C*, *A*, *h*) and whole-body black (*C*, *a*, *H*) rat coat-colour phenotypes, respectively (Supplementary Figs 9 and 10).

## Discussion

In this study, using the CRISPR/Cas platform in rats, we successfully recovered three distinct coat-colour phenotypes: albino (*c*) a missense mutation in the *Tyr* gene, non-agouti (*a*) a 19-bp deletion in the *Asip* gene and hooded (*h*) an integration of a 7,098-bp ERV elements in the *Kit* gene (Supplementary Fig. 1). Yang *et al.*^27^ have recently reported CRISPR/Cas-mediated genome editing in mice, including multiplexed targeted KOs, and a precise SNP exchange using ssODN donors^26^. They also created reporter and conditional KI alleles with site-specific insertions of a short Tag or a long fluorescent reporter, and double insertions of two loxP sites (floxed alleles), respectively^27^. In rats, three papers have been reported for CRISPR/Cas-mediated-targeted KOs^12^,^28^,^29^, and a more recent paper^38^ generated targeted KIs of floxed alleles using double-strand DNA (dsDNA) donor plasmids. However, accurate KIs with ssODNs, such as that reported in mice^26^,^27^, have not yet been demonstrated. To our knowledge, this is the first report to demonstrate that the CRISPR/Cas system can be used for accurate KI targeting with ssODNs in rats, thereby recovering disease-associated phenotypes of various types of mutations, such as SNPs, indels and large genomic structural variations.

Using the CRISPR/Cas system with ssODNs, we surprisingly detected the high efficiency of HDR-mediated KIs in rats. In mice, the high efficiency of HDR-mediated KIs has also been reported with ssODN donors^26^,^27^,^33^, compared with those of KIs with dsDNA donor vectors^39^. One of the major differences between ssODNs and dsDNAs is the lengths of the inserted DNA, such as 1–35 bp versus several hundred base pairs, respectively. The larger DNA fragment might be more difficult to integrate into target sites via HDR. Differences in the underlying mechanisms for HDR-based repair of CRISPR-mediated DSBs between ssODN and dsDNA donors might also serve as an explanation for the efficiency differences, although the exact repair mechanisms are still unknown^40^,^41^. Although the CRISPR/Cas system with ssODN donors in rats allowed us to mediate various kinds of genome editing, such as SNP exchange, site-specific insertion of short DNA fragment and a precise large DNA deletion, multiple cleavages to investigate large-scale chromosomal rearrangements is still a challenging task^42^,^43^.

Our CRISPR/Cas platform also facilitated allele-specific genome editing. The allele-specific CRISPR/Cas targeted the exact allele of the target gene by a specific gRNA that could discriminate a single SNP of the targeted allele in heterozygous F1 rat hybrids. In the Cas9/gRNA-injected embryos, the specific gRNA for the F344-albino mutation (gRNA:*Tyr*^*c*^) only targeted the F344-allele (6/21), while the gRNA for DA-wild-type (gRNA:*Tyr*^*C*^) only targeted the DA-allele (7/23), thereby changing the dominant coat-colour phenotype of the F1 pups (Fig. 2c). The allele-specific genome editing may be used for targeting genes that affect disease-related phenotypes in a dominant-negative manner. It may also be applicable to larger heterogeneous populations in which it has proven difficult to create inbred lines, or in human/primate cell lines or stem cells that are normally genetically very heterogeneous. Our F1-hybrid experiments have uncovered several additional findings that have not been observed in homozygous inbred strains so far. The CRISPR/Cas-mediated F1 pups showed complete or mosaic albino phenotypes (Fig. 2c). This indicates that the injected Cas9/gRNA carries out gene editing at the one-cell stage for changing the complete phenotype and at the two-cell stage (or later) for the mosaic phenotype, since in F1 hybrids only one target-allele exists at the one-cell stage, although there is the possibility that cleavage and/or repair occurred later in the developmental stage. The fact that the resulting CRISPR/Cas-mediated Wistar rat carried four mutations (Supplementary Fig. 3), which were all faithfully transmitted through the germline (Supplementary Table 1), also supports these findings. Furthermore, although we injected gRNA:*Tyr*^*c*^ (for F344) into the DA-derived male pronucleus, the F344-allele of the female pronucleus was modified in the embryos, suggesting that the CRISPR/Cas-mediated gene editing presumably occurred after the fusion of haploid gametes in the fertilized eggs.

Another novel finding allows conclusions regarding the mechanistic level of the process that is initiated by the initial DSB caused by the CRISPR/Cas system. The occurrence of albino coat-colour rats that carry homozygous alleles of F344 *Tyr*^*c*^but were derived by injection of gRNA:*Tyr*^*C*^ into *Tyr*^*C*^*/ Tyr*^*c*^ F1-hybrid embryos, suggests that interallelic gene conversion has occurred (Supplementary Fig. 8). The gRNA:*Tyr*^*C*^ and Cas9 induced DSBs at the targeted DA *Tyr*^*C*^ allele. This DSB was repaired using the F344 *Tyr*^*c*^allele as the template, resulting in an interallelic gene conversion between homologous chromosomes. Sequence analysis of the F1 rats with polymorphic SNPs between F344 and DA revealed that the interallelic conversion occurred beyond the 2-kb region of the targeted site, but did not exceed the 30-kb region around the site (Supplementary Fig. 11). These findings provide an explanation as to how CRISPR/Cas can mediate targeted homozygous mutations. The initially generated KO allele in the haploid nucleus is used as the template for repairing the subsequently mediated KO allele in the fused nuclei, causing targeted homozygous alleles in the one-cell stage embryo. These observations also suggest that CRISPR/Cas can be used for allele-specific gene therapy by targeted gene silencing or targeted gene conversion between homologous chromosomes to repair the dominant disease-related allele.

The frequency of OT mutations remains one of the biggest unknown variables concerning the use of CRISPR/Cas to modify genomes^20^,^34^,^44^. In this study, genomic loci containing up to three or four base-pair mismatches compared with the 20-bp gRNA sequences were amplified by PCR. The most likely 7 OT sites of gRNA:*Tyr* in 7 founder rats, 11 OTs of gRNA:*Asip* in 11 founder rats, 4 OTs of gRNA:*Kit-1* in 11 founder rats and 5 OTs of gRNA:*Kit-2* in another 11 founder rats were investigated (Supplementary Tables 2 and 3). No indel mutations at any of the examined potential OT sites were identified by sequence analysis in the all CRISPR/Cas-mediated founder rats (Supplementary Table 2). However, OT cleavages by CRISPR/Cas have been reported in several individual studies using human and rodent cells^20^,^44^. In general, the OT effects of CRISPR/Cas, which depend on the 20-bp target sequences of the gRNA and the terminal NGG protospacer adjacent motif sequences, seem to be higher than those of ZFNs and TALENs, which depend on the 30- to 36-bp target sequences detected by the two engineered proteins^30^. The double Cas9-nickase approach using the enhanced cleavage specificity with 40-bp of double-target sequences has been reported to reduce OT mutations^20^,^45^. Our *in vitro* study using Rat-1 cells suggested higher cleavage specificity of CRISPR/Cas than that of TALENs against the *albino* mutation (Fig. 2b). General comparisons among the three most recently used gene modifying systems (that is, ZFN, TALEN and CRISPR/Cas) in terms of their efficiency and specificity are not easy to make and can only be determined from case-by-case studies. It is difficult to draw comparisons between TALEN and CRISPR/Cas using our particular approach, since the targeted mutation is located at the 3′ critical sequences for gRNA binding^20^,^44^, but is not important for TALE binding at the 5′ sequence (Supplementary Fig. 12). The higher specificity of CRISPR/Cas in this study might be explained by the specificity of its DNA–RNA binding compared with that of the DNA–protein binding in the ZFN/TALEN systems. In our experiments, sequence analysis of Rat-1 cell DNA also revealed the presence of 5–8% mismatched cleavages even with the allele-specific gRNA (Fig. 2d; Supplementary Figs 4 and 5), while no mismatch cleavages were detected in the F1 rat embryos (Fig. 2g; Supplementary Figs 7 and 8). The difference in the cleavage specificity between the *in vitro* cells and the *in vivo* embryos, even using the same target sequences, might have been caused by differences in the protocols that were used. The DNA plasmids that constitutively express gRNA and Cas9 mRNA were transferred into Rat-1 cells, while the gRNA and Cas9 mRNA that transiently translate the protein were injected into rat embryos, resulting in shorter cleavage duration at lower concentrations in embryos compared with that in cells. Several *in vivo* studies reported in mice^26^,^27^, rats^12^,^28^,^29^ and other animals^21^,^22^,^23^ suggests less OT cleavages compared with those in *in vitro* studies^20^,^34^,^44^, thereby supporting our findings. In contrast to cell-based experiments using any of the above described nucleases, potential OT effects are normally crossed-out in animal studies. Every backcross generation removes potential off-targeted modifications by 50%, which means that only two times backcrossing clears already 75% of the genome from unintentional mutations that might have been induced by OT effects. Provided a careful selection of the target region and sophisticated design of the gRNA and considering a natural *de novo* mutation rate of 70–175 newly acquired mutations per diploid genome between two generations^46^,^47^, the number of OT mutations induced by nucleases only plays a minor role in this context.

In conclusion, we have shown that the CRISPR/Cas system provides effective genome editing in rats, such as KOs, KIs, allele-specific manipulations, gene conversion and the recovery of disease-related mutations. This powerful and efficient genome-editing technology can be used for creating animal models of many important human diseases as well as for prospective gene therapy approaches.

## Methods

### Animals

F344/Stm (NBRP-Rat No.0140), DA/Slc (NBRP-Rat No.0157) and PVG/Seac (NBRP-Rat No.0080) rats were provided by the National Bio Resource Project for the Rat in Japan ( www.anim.med.kyoto-u.ac.jp/nbr). Jcl:Wistar rats were obtained from CLEA Japan Inc. (Tokyo, Japan). The rats were kept under conditions of 50% humidity and a 14:10-h light: dark cycle. They were fed a standard pellet diet (F-2, Oriental Yeast Co. Ltd, Tokyo, Japan) and tap water *ad libitum*. Animal care and experiments conformed to the Guidelines for Animal Experiments of Kyoto University, and were approved by the Animal Research Committee of the Kyoto University.

### Cell culture and transfection by electroporation

Rat-1 cells were obtained from the RIKEN BRC Cell Bank (Tsukuba, Japan, www.brc.riken.jp/lab/cell/english). The cells were cultured in Dulbecco’s modified Eagle’s medium (Life Technologies, Carlsbad, CA, USA), supplemented with 10% fetal bovine serum in a humidified atmosphere containing 5% CO_2_ at 37 °C. REFs were isolated from the E14.5 embryos of F344 and DA rats. The REFs were cultured in Dulbecco’s modified Eagle’s medium supplemented with 10% fetal bovine serum. The Rat-1 cells and REFs (1 × 10^5^) were suspended in 10 μl of R buffer (supplied as part of the Neon Transfection System, Life Technologies) and each given 0.5 μg of the Cas9 and gRNA plasmid, after which they were electroporated using the Neon Transfection System (Life Technologies) under the following conditions: pulse voltage, 1,300 V; pulse width, 20 ms; and pulse number, 2. The *in vitro* transfer experiment was replicated three times.

### Plasmids expressing codon-optimized Cas9 and gRNA

Plasmid vectors expressing Cas9 and gRNA with the U6 promoter for transfection by electroporation (hCas9: ID#41815, and gRNA cloning vector: ID#41824, respectively), and with the T7 promoter for *in vitro* transcription (pMLM3613: ID#42251, and pDR274: ID#42250, respectively), were obtained from the Addgene repository ( www.addgene.org/CRISPR). To design the gRNAs, software tools (crispr.genome-engineering.org) predicting unique target sites throughout the rat genome were used. Oligonucleotides designed for target sites were cloned into an *Afl*II-digested gRNA empty vector or into *Bsa*I-digested pDR274. The target sites in the rat genome and the sequences of the ssODNs are shown in Supplementary Figs 12–14.

To prepare Cas9 mRNA and gRNA, T7-promoter Cas9 and gRNA expression plasmids were linearized with *Xho*I and *Hind*III, respectively, and extracted with NucleoSpin Gel and PCR Clean-up kits (Macherey-Nagel, Düren, Germany). After purification of the linearized DNA, Cas9 and gRNA mRNA were transcribed *in vitro* using a MessageMAXT7 ARCA-Capped Message Transcription Kit (CELLSCRIPT, Madison, WI, USA) and a MEGAshortscript T7 Kit (Life Technologies), respectively. Cas9 mRNA was then polyadenylated using a A-Plus Poly(A) polymerase tailing kit (CELLSCRIPT). The resultant mRNA was purified using a MEGAClear kit (Life Technologies) and resuspended in RNase-free water.

### Rat embryo microinjections

F344 and Wistar females of 8–12 weeks of age were superovulated by injection with gonadotropin serum from pregnant mares (PMSG: Aska Pharmaceutical Co., Tokyo, Japan) and human chorionic gonadotropin (hCG: Aska Pharmaceutical Co.). Then, pronuclear-stage embryos were collected from the superovulated females previously mated with males. They were cultured in a modified Krebs-Ringer bicarbonate solution^48^ before and after the microinjections^49^. Using a micromanipulator (Narishige, Tokyo, Japan), 100 ng μl^−1^ Cas9 mRNA and 50 ng μl^−1^ gRNA were microinjected into the male pronuclei of the embryos. For the KI experiments, 50 ng μl^−1^ ssODN was co-injected. Injected embryos were cultured in modified Krebs-Ringer bicarbonate medium overnight and the divided two-cell embryos were transferred into pseudopregnant Wistar females.

After microinjection, two-cell embryos were also collected. Genomic DNA was amplified with the GenomePlex Single Cell Whole Genome Amplification Kit (Sigma Aldrich, St Louis, MO, USA). After purification of the DNA, CRISPR/Cas-mediated mutations at target sites were analysed by direct sequencing.

### Cel-I nuclease assay and DNA sequence analysis

For the Cel-I nuclease assay to detect CRISPR/Cas9-mediated mutations, the SURVEYOR Mutation Detection Kit (Transgenomic, Omaha, NE, USA) was used in accordance with the manufacturer’s protocol. Briefly, 72 h after electroporation, genomic DNA was extracted from the Rat-1 cells and REFs using Nucleospin Tissue XS (Macherey-Nagel). PCR was then performed with the primers shown in Supplementary Figs 12–14. PCR amplification products were denatured and digested by the Cel-I nuclease, and then subjected to agarose gel electrophoresis.

For DNA sequence analysis, the PCR products were subcloned into a pCR4Blunt-TOPO plasmid vector (Life Technologies). Plasmids were extracted from the resultant *Escherichia coli* colonies for DNA sequencing. Sequencing was performed using a BigDye Terminator Cycle Sequencing Kit and an ABI PRISM 3130 Genetic Analyzer (Life Technologies).

### OT analysis

The potential OT sites in the rat genome (rn5) were identified using the latest version of the CRISPR design tool (crispr.mit.edu). All the potential sites were ranked by the OT hit score based on the predicted specificity^34^. To keep the OT analysis reasonable in the context of this study, ~10 potential sites were investigated per modified locus. Since a fixed OT score across all loci would have resulted in either too few or too many potentially examined OT sites, different scores were determined as follows: high-ranked potential sites (gRNA:*Tyr*^*c*^ and gRNA:*Asip*^*a*^;>0.8, gRNA:*Kit*^*h*^*-1*;>0.7, gRNA:*Kit*^*h*^*-2*;>0.3) were sequenced for OT analysis in the founder rats (Supplementary Tables 2 and 3).

## Author contributions

T.M. designed the work, produced all the data and wrote the paper. K.Y. performed the animal breeding experiments, cell culture, PCR and sequence analyses. T.K. and B.V. performed microinjection of CRISPR/Cas into rat embryos. All authors have read and edited the manuscript before submission.

## Additional information

**How to cite this article:** Yoshimi, K. *et al.* Allele-specific genome editing and correction of disease-associated phenotypes in rats using the CRISPR–Cas platform. *Nat. Commun.* 5:4240 doi: 10.1038/ncomms5240 (2014).

## Supplementary Material

Supplementary Information Supplementary Figures 1-14 and Supplementary Tables 1-3

## Figures and Tables

**Table 1:** CRISPR/Cas-mediated genome editing in rat embryos.

Injected RNA	Embryos injected	Two-cell embryos (%)	PCR-amplified (%)	Knockout (%)	Knock-in (%)
Cas9+gRNA	90	41 (45.6)	34 (82.9)	14 (41.2)	–
Cas9+gRNA+ssODN	91	46 (50.5)	38 (82.6)	5 (13.2)	14 (36.8)

**Table 2:** CRISPR/Cas-mediated genome editing in F1 hybrids.

Microinjection	Embryos injected*	Two-cell embryos (%)	Pups delivered (%)	*Tyr*^*c*^-KO (%)	*Tyr*^*C*^-KO (%)
Cas9+gRNA:*Tyr*^*c*^	104	48 (46.2)	21 (43.8)	6 (28.6)	0 (0)
Cas9+gRNA:*Tyr*^*C*^	135	71 (52.6)	23 (32.4)	0 (0)	7 (30.4)

^*^F1 embryos collected from the superovulated F344 female previously mated with DA males.

**Table 3:** CRISPR/Cas-mediated genome editing with ssODNs in F344 rats.

**Microinjection**	**Embryos injected**	**Two-cell embryos (%)**	**Pups delivered (%)**	***Tyr***		***Asip***		***Kit***		
				**KO (%)**	**KI (%)**	**KO (%)**	**KI (%)**	**Kit-1 KO (%)**	**Kit-2 KO (%)**	**KI (%)**
Cas9+gRNA:*Tyr*^*c*^+ssODN:*Tyr*^*C*^	94	45 (47.9)	13 (28.9)	3 (23.1)	1 (7.7)	–	–	–	–	–
Cas9+gRNA:*Asip*^*a*^+ssODN:*Asip*^*A*^	195	91 (46.7)	33 (36.3)	–	–	5 (15.2)	6 (18.2)	–	–	–
Cas9+gRNA:*Kit*^*h*^*-1*+gRNA:*Kit*^*h*^*-2*+ssODN:*Kit*^*H*^	104	43 (41.3)	25 (58.1)	–	–	–	–	9 (36.0)	0 (0)	1 (4.0)
