# Supplementary material for: Allele-specific genome editing and correction of disease-associated phenotypes in rats using the CRISPR–Cas platform
Source: Nat Commun. 2014 Jun 26;5:4240. doi: 10.1038/ncomms5240 (PMC4083438; doi:10.1038/ncomms5240)
Supplement: Supplementary Information — Supplementary Figures 1-14 and Supplementary Tables 1-3 [file ncomms5240-s1.pdf]

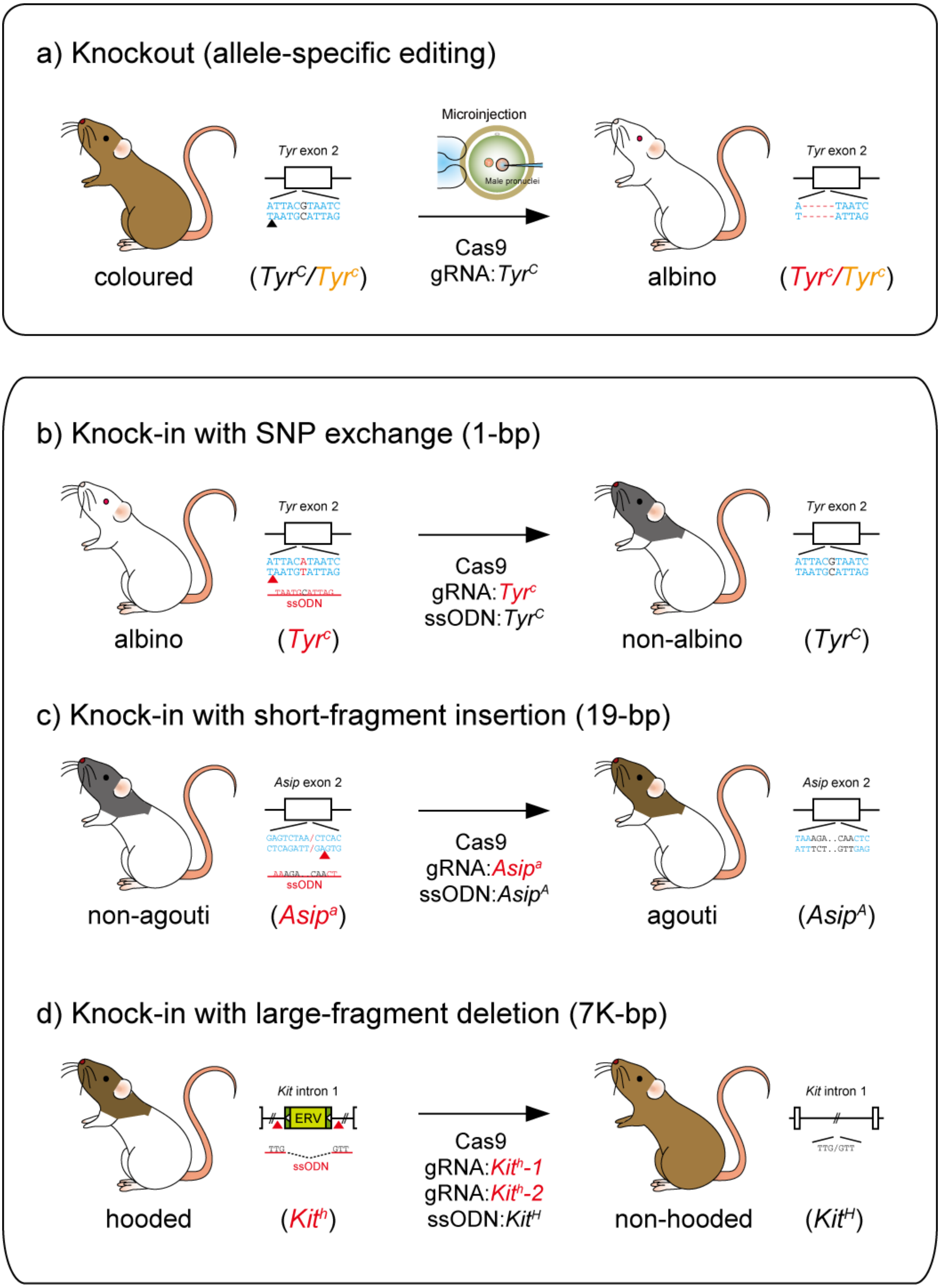

Supplementary Figure 1. Schematic representation of disease-specific genome editing in rats by CRISPR/Cas-mediated KO and KI. Our CRISPR/Cas platform in rats allowed: a) allele-specific genome editing for the dominant phenotype (KO), b) SNP exchange for the recessive *albino* phenotype (1-bp KI), c) integration of a short DNA fragment for the *non-agouti* phenotype (19-bp KI), and d) elimination of a large retrovirus sequence of the *hooded* phenotype (≈7Kb KI).

CRISPR-mediated mutations in Rat-1 cells

```
      CATGGTTTCCAGGATTATGTAATAGTGGTCCCT
   +2 CATGGTTTCCAGGATTATGTAAAATAGTGGTCCCT
   +1 CATGGTTTCCAGGATTATGTAAATAGTGGTCCCT
   +1 CATGGTTTCCAGGATTATGTAATTAGTGGTCCCT
   +1 CATGGTTTCCAGGATTATGTAAGTAGTGGTCCCT
   -3 CATGGTTTCCAGGATTAT---ATAGTGGTCCCT
   -4 CATGGTTTCCAGGATTATGTAA----GGTCCCT
   -5 CATGGTTTCCAGGATTAT-----AGTGGTCCCT
   -9 CATGGTTTCCAGG---------TAGTGGTCCCT
  -15 CATGGTTTCCA---------------GGTCCCT
-16+1 CATGGTTTCC---------------GGGTCCCT
  -23 CA-----------------------TGGTCCCT
  -24 CATGGTTTC------------/------CAGGT
  -25 CATGGTTTCC-----------/------TTCCA
  -30 TTGG-----------------/--GTGGTCCCT
  -33 CATGGTTTCCA----------/------TCACA
  -40 CATGGT---------------/------ACATA
  -42 ---------------------/------CATCA
```

30/95 (31.6%) clones, ave. 12.6 ± 2.8 bp

Supplementary Figure 2. Targeted *Tyr* gene disruption by CRISPR/Cas in Rat-1 cells. Plasmids expressing gRNA and codon-optimized Cas9 were transfected into the cultured rat fibroblast-like cell line, Rat-1. Sequence analysis of 95 colonies picked from the subcloned PCR products from the Rat-1 cells revealed that 30 colonies (31.6%) had various mutations (average mutation size, 12.6 ± 2.8 bp) at the targeted *Tyr* locus, as shown in red letters.

CRISPR-mediated mutations in Wistar rats

Wistar		CATGGTTTCCAGGATTATGTAATAGTGGTCCCT
#1-1	+1	CATGGTTTCCAGGATTATGTAAATAGTGGTCCCT
#1-2	+3	CATGGTTTCCAGGATTATTATATTATAGTGGTCCCT
	+2	CATGGTTTCCAGGATTATGTAAAATAGTGGTCCCT
	-3	CATGGTTTCCAGGATTATG---TAGTGGTCCCT
	-5	CATGGTTTCCAGGATTAT-----AGTGGTCCCT
#1-3	-1	CATGGTTTCCAGGATTATGTA-TAGTGGTCCCT

Supplementary Figure 3. NHEJ-mediated knockout by CRISPR/Cas in Wistar rats. Cas9 mRNA and gRNA were microinjected into the male pronucleus of fertilized Wistar rat embryos. Sequence analysis of the three pups delivered showed a variety of indel mutations at the targeted *Tyr* locus, as shown in red letters.

Cas9/gRNA:*Tyr*^{c}-mediated mutations in F344 REFs

```
    CATGGTTTCCAGGATTATGTAATAGTGGTCCCT
 +3 CATGGTTTCCAGGATTATGTAACCATAGTGGTCCCT
 +3 CATGGTTTCCAGGATTATGTATCCATAGTGGTCCCT
 +3 CATGGTTTCCAGGATTATGTAGTGATAGTGGTCCCT
 +1 CATGGTTTCCAGGATTATGTAAATAGTGGTCCCT
 +1 CATGGTTTCCAGGATTATGTAATTAGTGGTCCCT
 +1 CATGGTTTCCAGGATTATGTAACTAGTGGTCCCT
 -2 CATGGTTTCCAGGATTATGT--TAGTGGTCCCT
 -2 CATGGTTTCCAGGATTATGTA--AGTGGTCCCT
 -3 CATGGTTTCCAGGATTATGTAA---TGGTCCCT
 -4 CATGGTTTCCAGGATTAT----TAGTGGTCCCT
-14 CATGGTTT--------------TAGTGGTCCCT
-45 CTTTG----------------/------TCACA
-75 TGGGG----------------/------CTATT
-75 CCTGC----------------/------GGCTA
-94 GGAGC----------------/------CTGAT
```

42/88 (47.7 ± 6.1%) clones, ave. 11.7 ± 4.4 bp

Cas9/gRNA:*Tyr*^{C}-mediated mutations in F344 REFs

```
    CATGGTTTCCAGGATTATGTAATAGTGGTCCCT
 -7 CATGGTTTCCAGGATTATGTAA-------CCCT
 -8 CATGGTTTCCAGGAT--------AGTGGTCCCT
-13 CATGGTTTCCAG-------------TGGTCCCT
```

4/91 (4.4 ± 0.1%) clones, ave. 8.8 ± 1.4 bp

Supplementary Figure 4. Allele-specific genome editing in REFs derived from F344 rats. Plasmids expressing Cas9 and allele-specific gRNA, gRNA:*Tyr*^{c} and gRNA:*Tyr*^{C}, were transfected into cultured REFs derived from F344 rats. Sequence analysis of the colonies picked from the subcloned PCR products from the F344 REFs showed a variety of mutations at the targeted *Tyr* locus, as shown in red letters.

Cas9/gRNA:*Tyr*^{c}-mediated mutations in DA REFs

```
      CATGGTTTCCAGGATTACGTAATAGTGGTCCCT
   +3 CATGGTTTCCAGGATTACGTAAAAATAGTGGTCCCT
   +2 CATGGTTTCCAGGATTACGTAACATAGTGGTCCCT
   +1 CATGGTTTCCAGGATTACGTAATTAGTGGTCCCT
   +1 CATGGTTTCCAGGATTACGTAATTAGTGGTCCCT
  -25 CATGGTTTCCAGGATTA----/------CATCA
```

6/94 (6.4 ± 1.2%) clones, ave. 6.0 ± 4.9 bp

Cas9/gRNA:*Tyr*^{C}-mediated mutations in DA REFs

```
      CATGGTTTCCAGGATTACGTAATAGTGGTCCCT
   +5 CATGGTTTCCAGGATTACGTAATCTCAGAGTGGTCCCT
   +2 CATGGTTTCCAGGATTACGTAATTTAGTGGTCCCT
   +2 CATGGTTTCCAGGATTACGTAAAATAGTGGTCCCT
   +1 CATGGTTTCCAGGATTACGTAAATAGTGGTCCCT
   +1 CATGGTTTCCAGGATTACGTAATTAGTGGTCCCT
   +1 CATGGTTTCCAGGATTACGTAACTAGTGGTCCCT
   -1 CATGGTTTCCAGGATTACGTAA-AGTGGTCCCT
   -1 CATGGTTTCCAGGATTACGTA-TAGTGGTCCCT
   -3 CATGGTTTCCAGGATTACGTA---GTGGTCCCT
   -5 CATGGTTTCCAGGATTA-----TAGTGGTCCCT
   -6 CATGGTTTCCAGGATTAC------GTGGTCCCT
-11+5 CATGGTTTCCAGGA--ACACC----TGGTCCCT
-17+1 CATGGTTTCCAGGATT----------------G
  -18 CATGGTTTCCAGGATT-----/------AGGTG
  -23 CATGGTTTCCAGGT-------/------TGTTC
  -24 CATGGTTTCCAGG--------/------TGTTC
  -32 CAT------------------/------GGTGT
  -43 GGGTT----------------/------CAGGT
  -74 GGGAG----------------/------CCTGA
```

35/93 (37.6 ± 3.3%) clones, ave. 9.2 ± 3.3 bp

Supplementary Figure 5. Allele-specific genome editing in REFs derived from DA rats. Plasmids expressing Cas9 and allele-specific gRNA, gRNA:*Tyr*^{c} and gRNA:*Tyr*^{C}, were transfected into cultured REFs derived from DA rats. Sequence analysis of the colonies picked from the subcloned PCR products from the DA-REFs showed a variety of mutations at the targeted *Tyr* locus, as shown in red letters.

TALEN-mediated mutations in F344 REFs

```
       AGGATTATGTAATAGTGGTCCCTCAGGTGTTCCATCACATAAAACCTGATGGCTATTATA
  -1   AGGATTATGTAATAGTGGTCCCTCAGGTGTCCCATCACATAAAACCTGATGGCTATTATA
  -3   AGGATTATGTAATAGTGGTCCCTCAGGTGTTC---CACATAAAACCTGATGGCTATTATA
-5+1   AGGATTATGTAATAGTGGTCCCTCAGGTG--C--TCACATAAAACCTGATGGCTATTATA
  -5   AGGATTATGTAATAGTGGTCCCTCAG-----CCATCACATAAAACCTGATGGCTATTATA
  -5   AGGATTATGTAATAGTGGTCCCTCAGGTGTTCCATC-----AAACCTGATGGCTATTATA
  -6   AGGATTATGTAATAGTGGTCCCTC------TCCATCACATAAAACCTGATGGCTATTATA
  -6   AGGATTATGTAATAGTGGTCCCTCAGG------ATCACATAAAACCTGATGGCTATTATA
  -7   AGGATTATGTAATAGTGGTCCCTCAGG-------TCACATAAAACCTGATGGCTATTATA
  -8   AGGATTATGTAATAGTGGTCCCTC--------CATCACATAAAACCTGATGGCTATTATA
-9+4   AGGATTATGTAATAGTGGTCCCTCA--TGT-C--TCACATAAAACCTGATGGCTATTATA
  -9   AGGATTATGTAATAGTGGTCC---------TCCATCACATAAAACCTGATGGCTATTATA
  -9   AGGATTATGTAATAGTGGTCCCTCA---------TCACATAAAACCTGATGGCTATTATA
 -10   AGGATTATGTAATAGTGGTCCCTCAGGTGT----------AAAACCTGATGGCTATTATA
 -10   AGGATTATGTAATAGTGGTCCCTCAGGT----------ATAAAACCTGATGGCTATTATA
 -14   AGGATTATGTAATAGTGGTCCCT--------------CATAAAACCTGATGGCTATTATA
```

24/85 (28.2 ± 4.6%) clones, ave. 6.5 ± 0.7 bp

TALEN-mediated mutations in DA REFs

```
       AGGATTACGTAATAGTGGTCCCTCAGGTGTTCCATCACATAAAACCTGATGGCTATTATA
  -2   AGGATTACGTAATAGTGGTCCCTCAGGTGT--CATCACATAAAACCTGATGGCTATTATA
-5+2   AGGATTACGTAATAGTGGTCCCTCAAC---TCCATCACATAAAACCTGATGGCTATTATA
  -4   AGGATTACGTAATAGTGGTCCCTCAG----TCCATCACATAAAACCTGATGGCTATTATA
  -4   AGGATTACGTAATAGTGGTCCCTCAGGTGT----TCACATAAAACCTGATGGCTATTATA
  -4   AGGATTACGTAATAGTGGTCCCTCAGGTGTTC----ACATAAAACCTGATGGCTATTATA
-5+1   AGGATTACGTAATAGTGGTCCCTCAGGTG--C--TCACATAAAACCTGATGGCTATTATA
  -5   AGGATTACGTAATAGTGGTCCCTCAGGTG-----TCACATAAAACCTGATGGCTATTATA
  -6   AGGATTACGTAATAGTGGTCCCTCA------CCATCACATAAAACCTGATGGCTATTATA
-8+2   AGGATTACGTAATAGTGGTCCCTCAG---TT---TCACATAAAACCTGATGGCTATTATA
  -7   AGGATTACGTAATAGTGGTCCCTCAGG-------TCACATAAAACCTGATGGCTATTATA
  -7   AGGATTACGTAATAGTGGTCCCTCAGGTG-------ACATAAAACCTGATGGCTATTATA
-9+2   AGGATTACGTAATAGTGGTCCCTCAGGTGT-CC------TAAAACCTGATGGCTATTATA
  -9   AGGATTACGTAATAGTGGTCCCT---------CATCACATAAAACCTGATGGCTATTATA
 -10   AGGATTACGTAATAGTGGTCCCTCAGGTG----------TAAAACCTGATGGCTATTATA
-11+1  AGGATTACGTAATAGTGGTCCCTCAGGTG---------A-AAAACCTGATGGCTATTATA
-12+1  AGGATTACGTAATAGTGGTCCCTCA--T---------CATAAAACCTGATGGCTATTATA
 -12   AGGATTACGTAATAGTGGTCCC------------TCACATAAAACCTGATGGCTATTATA
 -16   AGGATTACGTAATAGTGGTCCCTCAGG----------------ACCTGATGGCTATTATA
 -21   AGGATTACGTAATAGTGGTCCCTC---------------------CTGATGGCTATTATA
 -25   AGGATTACGTAAT-------------------------ATAAAACCTGATGGCTATTATA
 -31   AGGATTACGTAATAGTG-------------------------------ATGGCTATTATA
-36+3  AGGATTACGTAATAGTGGTCCCTCAGG-----/-------------------CTATTCTG
 -67   GTTTC---------------------------/----------------------TGATC
-120   TACTG---------------------------/---------------ATGGCTATTATA
```

31/94 (33.0 ± 0.9%) clones, ave. 15.4 ± 4.2 bp

Supplementary Figure 6. TALEN-mediated mutations in REFs. Plasmids expressing TALENs targeting the albino F344 allele (*Tyr*^{c}) were transfected into REFs derived from F344 rats and DA rats. Sequence analysis of the colonies picked from the subcloned PCR products from the REFs showed a variety of mutations at the targeted *Tyr* locus, as shown in red letters.

Cas9/gRNA:*Tyr*^{c}-mediated mutations in F1(F344XDA) rats

```
F1 hybrids   F344-allele   CATGGTTTCCAGGATTATGTAATAGTGGTCCCT
               DA-allele   CATGGTTTCCAGGATTACGTAATAGTGGTCCCT

      #1-2   F344-allele   CATGGTTTCCAGGATTATGTAAAATAGTGGTCCCT
               DA-allele   CATGGTTTCCAGGATTACGTAATTAGTGGTCCCT

      #1-4   F344-allele   CATGGTTTCCAGGATTATGTAATTAGTGGTCCCT
               DA-allele   CATGGTTTCCAGGATTACGTAATAGTGGTCCCT

                           CATGGTTTCCAGGATTATGTA---GTGGTCCCT
      #1-9   F344-allele   CATGGTTTCCAGGATTATGTAAATAGTGGTCCCT
               DA-allele   CATGGTTTCCAGGATTACGTAATAGTGGTCCCT

      #2-1   F344-allele   CATGGTTTCCAGGATTATGTAATAGCCAGGGATTTGGTCCCT
               DA-allele   CATGGTTTCCAGGATTACGTAATAGTGGTCCCT

      #2-6   F344-allele   CATGGTTTCCAGGATTATGTAATTTAGTGGTCCCT
               DA-allele   CATGGTTTCCAGGATTACGTAATAGTGGTCCCT

      #2-8   F344-allele   CATGGTTTCCAGGATTATGTAAATAGTGGTCCCT
               DA-allele   CATGGTTTCCAGGATTACGTAATAGTGGTCCCT
```

Supplementary Figure 7. Allele-specific genome editing with gRNA:*Tyr*^{c} in (F344 × DA)F1 hybrid rats. Cas9 mRNA and gRNA:*Tyr*^{c} were microinjected into the male pronucleus of fertilized (F344 × DA)F1 hybrid embryos. Sequence analysis of DNA from the pups delivered showed a variety of indel mutations at the targeted *Tyr*^{c} allele, as shown in red letters.

Cas9/gRNA:*Tyr*^{C}-mediated mutations in F1(F344XDA) rats

```
F1 hybrids   F344-allele   CATGGTTTCCAGGATTATGTAATAGTGGTCCCT
               DA-allele   CATGGTTTCCAGGATTACGTAATAGTGGTCCCT

      #1-1   F344-allele   CATGGTTTCCAGGATTATGTAATAGTGGTCCCT
               DA-allele   CATGGTTTCCAGGATTACGTAATTACGG(143bp)GTAATAGTGG

      #1-8   F344-allele   CATGGTTTCCAGGATTATGTAATAGTGGTCCCT
               DA-allele   CATGGTTTCCAGGATTACGTAAAATAGTGGTCCCT

             F344-allele   CATGGTTTCCAGGATTATGTAATAGTGGTCCCT
      #2-1                 CATGGTTTCCAGGATTAC---TTAGTGGTCCCT
               DA-allele   CATGGTTTCCAGGATTACGTAAAATAGTGGTCCCT

      #2-2   F344-allele   CATGGTTTCCAGGATTATGTAATAGTGGTCCCT
               DA-allele   CATGGTTTCCAGGATTATGTAATAGTGGTCCCT

      #2-3   F344-allele   CATGGTTTCCAGGATTATGTAATAGTGGTCCCT
               DA-allele   CATGGTTTCCAGGATTACGTAAATAGTGGTCCCT

      #2-6   F344-allele   CATGGTTTCCAGGATTATGTAATAGTGGTCCCT
               DA-allele   CATGGTTTCCAGGATTACGT------GGTCCCT

      #2-8   F344-allele   CATGGTTTCCAGGATTATGTAATAGTGGTCCCT
               DA-allele   CATGGTTTCCAGGATTATGTAATAGTGGTCCCT
```

Supplementary Figure 8. Allele-specific genome editing with gRNA:*Tyr*^{C} in (F344 × DA)F1 hybrid rats. Cas9 mRNA and gRNA:*Tyr*^{C} were microinjected into the male pronucleus of fertilized (F344 × DA)F1 hybrid embryos. Sequence analysis of DNA from the pups delivered showed a variety of indel mutations at the targeted *Tyr*^{C} allele as shown in red letters. Albino coat-colour F1 rats (#2-2 and #2-8) carried homologous alleles of F344 *Tyr*^{c}, indicating an interallelic gene conversion between the F344 and DA alleles.

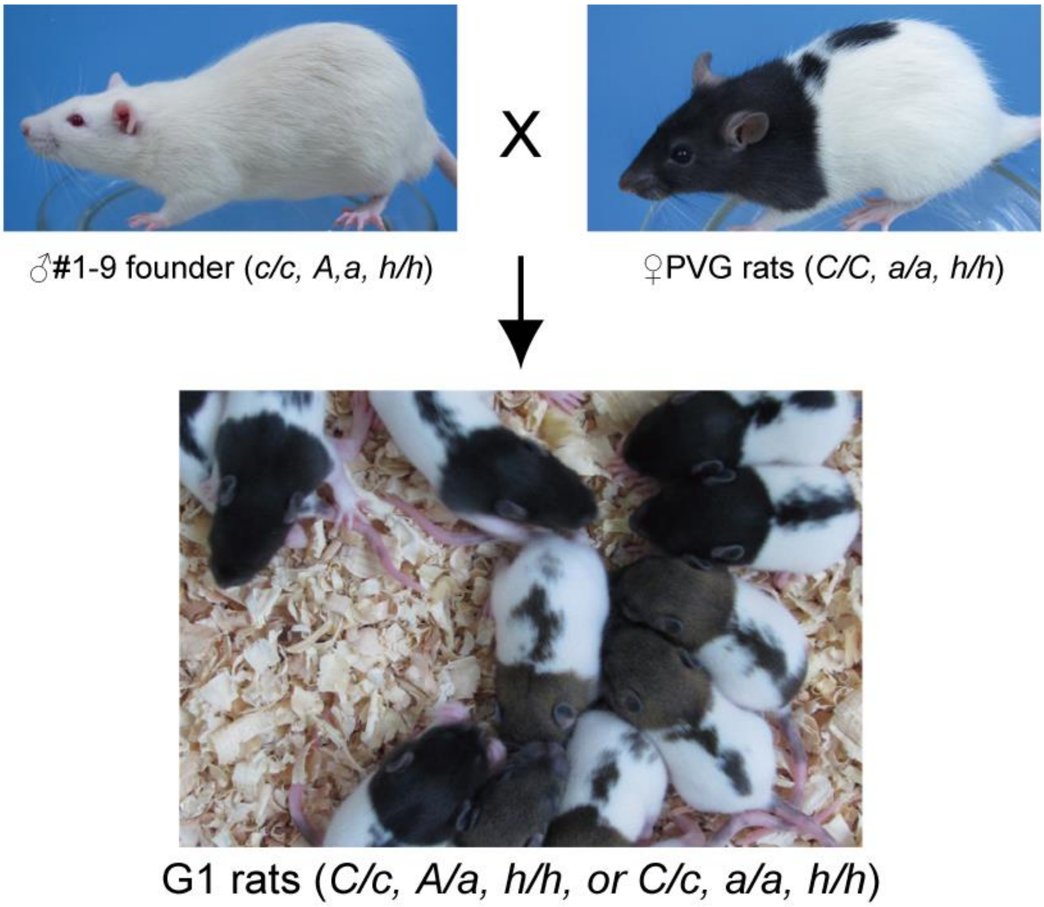

	#1-9 (G0)	PVG	G1	
Phenotype	*albino*	*black-hooded*	*agouti-hooded*	*black-hooded*
Genotype	Tyr^{c}/Tyr^{c}, Kit^{h}/Kit^{h}, $Asip^{A}$, $Asip^{a}$	Tyr^{C}/Tyr^{C}, Kit^{h}/Kit^{h}, $Asip^{a}/Asip^{a}$	Tyr^{C}/Tyr^{c}, Kit^{h}/Kit^{h}, $Asip^{A}/Asip^{a}$	Tyr^{C}/Tyr^{c}, Kit^{h}/Kit^{h}, $Asip^{a}/Asip^{a}$
Numbers	1	1	5	9

Supplementary Figure 9. Recovery of the coat-colour phenotypes for the *non-agouti* mutation by CRISPR/Cas. Crossing the CRISPR/Cas-mediated $Asip^{A}$ founder #2-6 (*c*, *A*, *h*) with *black-hooded* PVG/Seac rats (*C*, *a*, *h*) resulted in recovery of the coat-colour phenotype (black arrows), *Agouti-hooded* (*C*, *A*, *h*). This indicates that the integration of the 19-bp fragment mediated by CRISPR/Cas recovers the *agouti* from the non-*agouti* phenotype.

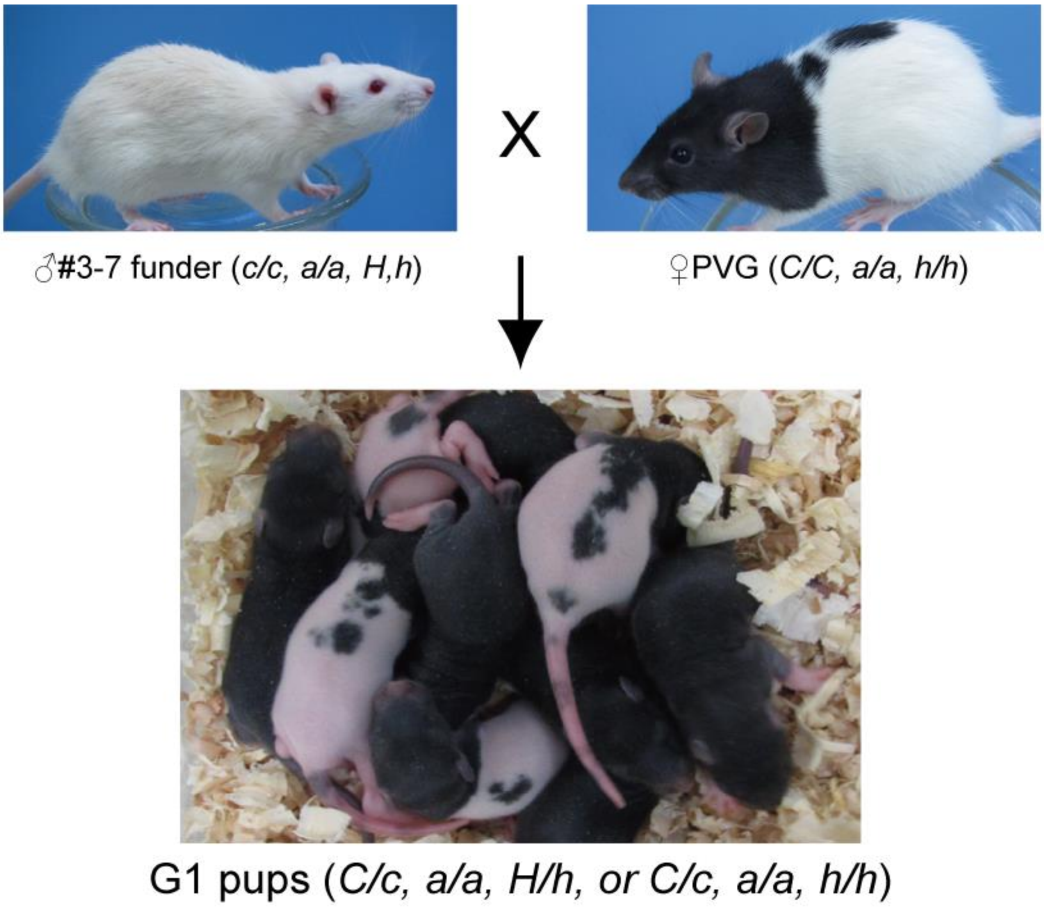

	#3-7 (G0)	PVG	G1	
Phenotype	*albino*	*black-hooded*	whole-body *black*	*hooded*
Genotype	Tyr^c/Tyr^c, $Asip^a/Asip^a$, Kit^H, Kit^h	Tyr^C/Tyr^C, $Asip^a/Asip^a$, Kit^h/Kit^h	Tyr^C/Tyr^c, $Asip^a/Asip^a$, Kit^H/Kit^h	Tyr^C/Tyr^c, $Asip^a/Asip^a$, Kit^h/Kit^h
Numbers	1	1	6	4

Supplementary Figure 10. Recovery of the coat-colour phenotypes for the *hooded* mutation by CRISPR/Cas. Crossing the CRISPR/Cas-mediated Kit^H founder #3-7 (*c*, *a*, *H*) with *black-hooded* PVG/Seac rats (*C*, *a*, *h*) resulted in recovery of the coat-colour phenotype, whole-body *black* (*C*, *a*, *H*). This indicates that the elimination of the 7,098-bp ERV fragment mediated by CRISPR/Cas recovers the *hooded* from the *non-hooded* phenotype.

12).

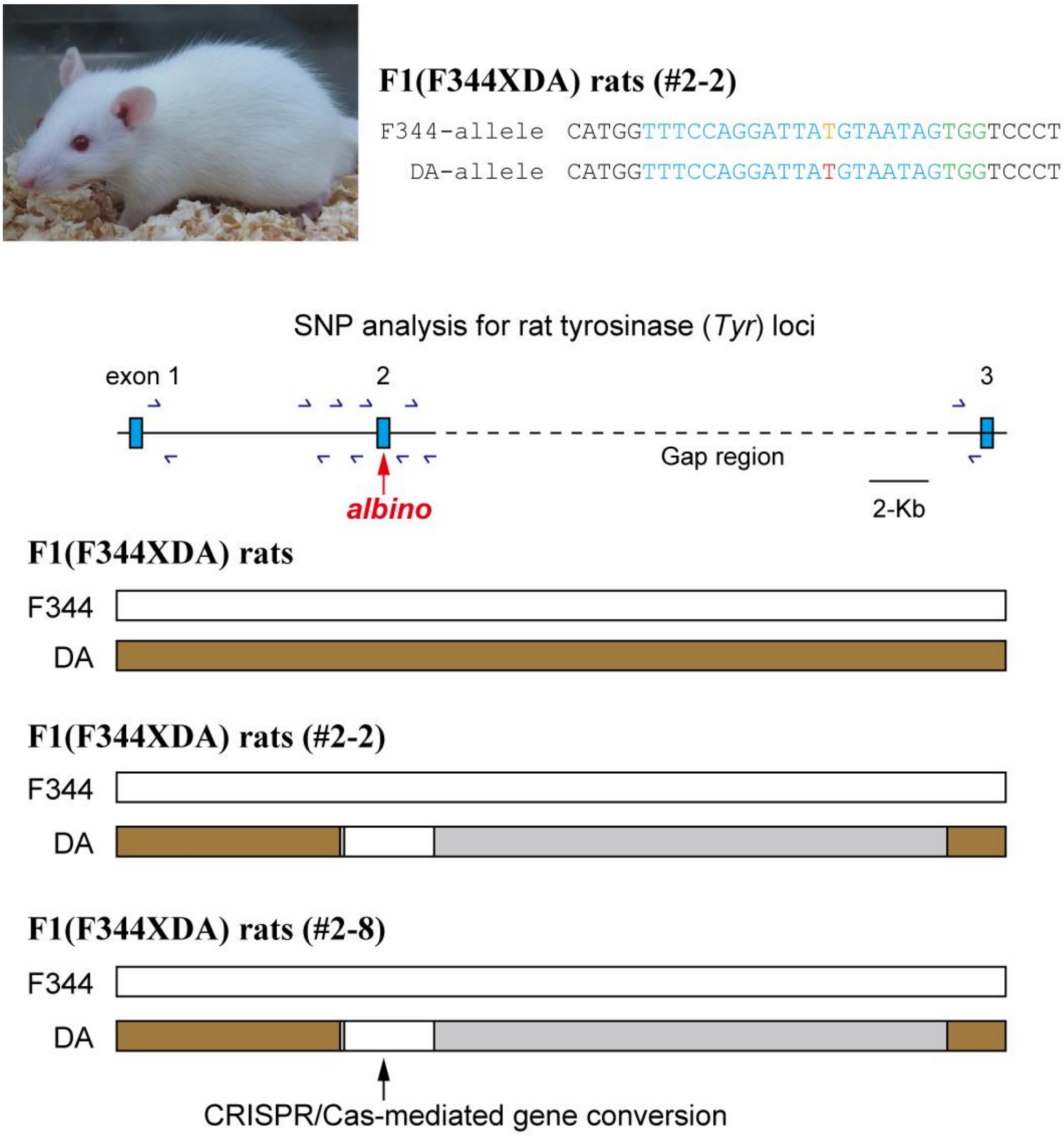

Supplementary Figure 11. CRISPR/Cas-mediated (F344 × DA)F1 rats (#2-2, #2-8) carrying homologous alleles of F344 *Tyr*^{c}. gRNA:*Tyr*^{C} and Cas9 induced DSBs at the targeted DA *Tyr*^{C} allele, which were repaired using the F344 *Tyr*^{c} allele as template, resulting in an interallelic gene conversion between homologous chromosomes. SNP analysis (with the primers represented by blue arrows) of the F1 rat DNA revealed that interallelic conversion (white box) occurred beyond the 2-Kb region of the targeted *albino* site, but within the 30-Kb region around the site (see also Supplementary Figure 12).

```
Tyr Up F
CACTCTTAAATGCAGCCAAGGACTATAAAGAGAAAGATAGAAAGAATGCATAAATCAGAGTTCTACATC
AAGCCCAGCGTCAGGGCTGGGCCAGTTAGGTTATTGTCAGTTTTACATAAACTTAGACAGACTCACGAA
GGGAAATCTCAGCTCAGTTTTTCTTACATAAGATTGCCACGTTGACTTTAATAGAGAACA/TCTTTTGG
TTGGTAATTGATATTAGAATGGTCAGTCCCATCTTTGGGCATATATCCATTGTAGTATAAAATGGAAAA
ACAGGCCTAAGTTTTGAGAATTAATCAGCTTGTTATTTTTGTCATGAAATTCTAAACAATTTTAGCTGT
GAATTTGTGTTCCATAGTGAAGCCATGGGGACAGGGCAG/ATTAACTATAGCAGAAATTTTACAAATTA
AC/TTCTATCTT/CAAATATTTATATAAAGTACTTACTTTCATCACAATTGAATGTGTATCACACAGGA
AACCTAAGTCATTAGATTGAGGACGGGAACGCCACCCTGGGTTTGACCTTCCATCCCTTGTCCTGCAAA
CTCTACTGCGTCCTTTGTCAGTGCTAAGAATGGTGGATATCCCACTTCATGCTTTTTTGGTTTTGTCAT
AACAGATCCATTTTTAATGTCTGGCTTGATATACAGCATAGTCTTTCCTTAGAGAATTTTACAACATAG
CAGTTTCTTAGATTCCTATGTTACCCTCTGGCAGATTATCATCATCATTATTTTTTT/-AAAATCACGA
ATCTTTACGTTTTATAGGAAGTATTTTAAGGAGTTACGTTGCACATATTTCGTTTAAGTCATGTGTTAG
TATTTTCCATATTGATAAGTAAACTGAAAGTCAGAGAAGGTAAATGACAGTTTGAAGATAAAAGACCTT
GCTTCCAGTACAAACCAAGTTTTTCAGCTCCAACTTTATTGTACCCTGTGCAGTTATTTGTTCATTTAC
TTTTACTGGCATGTGCATTATG/ATGCATGTGGCACACATTATATGCATGCAAACAAGTGTGTGTTTAT
ATGTTGATAGGTATATGGGTATCTACAAGTGTACTTGTACATGTACATGTTTGTGTGTGCCCATGGAGA
CCATAGGTCGACACTTAGTGTCTTTCTCAATTACTTACTATGGTACACATTAAGGCATGGTCTCTGTCT
               Tyr_Large_F                                    Tyr_Up_R
GACTGGATGGAATAACACTCATTCATATGACTAGTTTCCTTAGCCAGATTGCTCTGGCAATTTTCTGTT
TGCATTTTGCAAGCACCGGGATGCTAACAGGAATCTACTTCTCACATTCAGGAATTAAGAGGGTGCTGA
TTATGTGACTTCCAGTTCTTAAGTTGGCA/GTTGCAAGTATTTTACTCACTGAAACATTGCCCTTTTCT
GTCTCCATGCATTTATAACTTCCTGCAAAGGCGTTCAGTAGCCAATGTTTTCACAGA/CGCTTCTATGT
TAAAATGAACAACAGATTTCCAGTTCTTCATCTAGTTGGCCTACGTTTTTGGAAGTCAATGTGCAACAG
TGTGAAATAAGACTTGTTTATTTGAACAGAGGTGCTCATGTATTATGAAGTTTGTACACGGAAAAGAAA
AAGAAAGAAGAGAAAACAGACTGGACTAGTATGGTGACTTGGGAGCCTGTCTGGGAGGCAGAGTCACTA
CTGACAAGTTACTCAATGTCACATAAAAGAAATGTGGCTCCATTTTATGAATTATGACTAAAAATAGCT
CAAGGTTTAGTTGGGTACTTTCTGAACACAAATTAGACTAGCAAATGCCAGCTACAAGAACAATAAATG
TCTAGCAC/TGGTAGCCTAAGTTCCTCCCTTCACTTTCATCTAACTGCTAGGAAGCTGAATGATAATAA
TGATATGTAATTTCATTGTATGTAATTTCATTCATATGTAAGTCCCTTGTCTCAGAGATAACT/GCATT
TTGCATAAATTGGTTTTCACAGATCATTTGCAGCAGATCAGAAGAGTATAATAGCCATCAGGTTTATG
Tyr_Small_F           PAM TALEN_R   Target                     TALEN_L
TGATGGAACACCTGAGGGACCACTATTACA/GTAATCCTGGAAACCATGACAAAGCCAAAACCCCCAGG
CTCCCATCTTCAGCAGACGTGGAATTTTGTCTGAGTTTGACCCAGTATGAATCTGGATCAATGGATAGA
     Exon_2                                         Tyr Small R
ACTGCCAATTTCAGCTTTAGAAACACACTGGAAGGTAATATTTTCATGTTTAAATTTTTAAATTTTAAT
TTTTTT-/TCTTAC/TTTATAATTTCTTTTCAGAAATTCTGGGCTCAGAGATATTTTGTAATTTTGTGA
GTTTTGCAATGCTAAGCTTTGAATATACCCAAGGAGATAGTAAACCTAGCCAGCATAGAT/ATATCTCA
CTATATTCTAGAAGTGTAGTGATAGAAAAGTGTACTATACCCATGGCAAAGGTATGGCAC/ATTTTCCA
CAAAAACAAAATAAAGCAAAACAGAAAACTAAAACAATAACAAAAGTTTCAGTTCTCATCTCAAACGAA
GAAAAAAAGAAAAAGATAGATTCTCAAAAGCCAAAAGGGAGTGACCATGGTCAGAGTATTTTCCAATAG
                                                 Tyr_Large_R     Tyr Down F
TATTTTCCTTTTTTCTATTTAATTTATTTTTTATTGT/---TTTTTTTAGTTACATTTCAAATGTTATT
ACCTTTCCTCATTTCCC/-ATCCATAAGA/CCCCCACCCCT/CTCCCCCTCCCCCTTTTTTATAAGATT
ACTCCCCCTCCCCAACCACT/ACCCCTTTCCCTCCTCCTCACCTTGACATTTCCCTACACTGGGGGTCG
AGCCTTGGC/AAGGACCAAGGGGTTCTCCTTCATTTGGTGCCCAACAAGGCCACT/CCTCTGCTACATA
TGTAGCTGGAGCCATGAGTCAGTCCATGTATAGTCCTTGGGTAGTGGTTTAG/ATCCCTGGGAGCTCTA
G/ATTTGTTTA/GCATTGTTGTCCTTATGGGGTTGAAAGCCCCTT/CCAGCTCCTTCAATCCTTTTCTA
ACATGATAAGAGTTCTGTGAAGTCTTACAGTAATGGAAAAAAGAAAATCAAAGGTCAAGAAATATTACA
AGTTCATCAAAGGAAGTATCAAGTACGCAGGTTATAGAAAAGCCATGAGACCTAAGCTATAGGCATCCT
ATACCATCTGACCTCCATTCATAGCCTTTTCATTGTTCAAAAGTAGGAGACTATATATACAATATCATT
TATCCAATG/ACAATGTTAGTTTACATACAGAGGAAAGCAATAAGTGGCTATTTACCCAACATCTGCAC
TTCATCAGAGATAGTTTCTCGCATTTTTTCTATT---/ATTCCTTAATCCAGCACATGCTCACATTTTT
CTTTCAACACTAGTTTATTCATGTGCAATCAAAATGGATCCTTTCACATACCCATGCATTCAATTCATT
TAGAATTAGGGAAACAAATCTATCAATACACACACACACACACACACACACACACACACACACACACAA
GTTTTTTTTTTTTTTTATTAACTTGAGTANNNNNNNNNN                       Tyr_Down_R
```

Supplementary Figure 12. gRNA designed to target the *Tyr* locus and the primer sequences used for PCR analyses of the *Tyr* gene. The gRNA-binding sequences (blue) and the PAM sequences (green) are shown in exon 2 (red) of the *Tyr* locus. ssODN:*Tyr*^{C} sequences consisting of the G allele of *Tyr*^{C} are underlined. TALEN pairs designed to target the *Tyr*^{c} locus are shown by yellow boxes. The two sets of primers (small and large) used for the PCR analysis and the two sets of primers (up and down) used for the SNP analysis are shown in boxes. Polymorphic SNPs between the F344 and DA strains are highlighted in yellow (F344 allele in #2-2 and #2-8) and in brown (DA allele in #2-2 and #2-8) (see also Supplementary Figure 11).

Asip Large F
GAGGCCCTTTCCACAACTCTGCCTAGGCCTAAGGTCTCTGGGGACTGTTGCTTGTAC
AGGATGGGGTAAAAAAAGAAAGAGAAAGAAAAGAAACACAGAGAGTCAGTACTCCGC
CCTCTGGACAGCACAAAGCAACACAAAGAATTCTTGTCCCCTTGGCTTCAGTCTCCC
TCCTTCCCTCCCTCCCTCGTCTTTCTTCTCCCCATCTCTCCTTTTCTGTCTTCTCCC
CATATCAACATCCCTTCCCTACCCTCAGCCTTGCTCCACTTCCGGCCCTTCTGAGAG
ACTTGTGTGGCAGAGTCGGACCACCAGTCCAAAGGCACACAGAGAGAGCAAAAAGGT
ACATTCATCCCTTACCACCATCTTCTCTCCCACCCACCCCTGATCCCCTTCTCTGCC

Exon_2 *Asip* Small F
CAACTTAGGCTTCTCAGGATGGATGTCACCCGCTTACTCCTGGCCACCCTCGTGGGC
TTCCTGTGCTTCCTCACCGTCCACAGCCACCTGGTATTTGAGGAGACGCTTGGAGAT

Asip target 19-bp deletion PAM
GACAGGAGTCTAAAGAGCAACTCTTCCATCAACTCACTGGATTTCTCCTCTGTTTCC
ATTGTGGGTGAGTAGCCTGCCTTGGGACCCAGCCTCTAGGCTCTGACCCATGAGAGG
GAGTACAGTATGTCATCCTGCTGCCTGAGCTGCCTCCCGCCACAGCTATGGTGCTTT

Asip Small R
CTCCCTCATTCCTAGGGAAATAGCGTCCTTGGCAGTGTCCTTGAGAGCTTGAGTTTG
GGAAAGCTCTGCGCGCCACCACTGTAATACCAAGTTTAGTGTGTCAGAGATCTGAAG
AAGCCTTAGGTCAAGGTGCGTGCTTGGTGGACTTGGTTTTATTTAGCATTGATGAAC
TTTAACGAATCGTAGAATTCTTCAACAAAACAAACTTTAGGTAAACCTCGGAACTAG
AAATCAGTCCGCGATAAATGAGTCTGGCCACGGGATGGGAAGTTACGAGGAGTAGCC
GAGTCCGGCCTTGGAGATCTTGTGTGGTTTGATCTCACCTAATCATAAAGGGCTGGG
GATGAAG *Asip*_Large_R

Supplementary Figure 13. gRNA designed to target the *Asip* locus and the primer sequences used for PCR analyses of the *Asip* gene. The gRNA-binding sequences (blue) and the PAM sequences (green) are shown in exon 2 (red) of the *Asip* locus. ssODN:*Asip*^{A} sequences consisting of the 19-bp *Asip*^{a} deleted sequences (purple) are underlined. The two sets of primers (small and large) used for the PCR analysis are shown in boxes.

```
Intron1
     Kit-1 Large F
AATTCCTGGTCCAACACAGCCCAGTAAGGGTTCTTAATCGCTTTCCAGAACTGCTTCCAGACAGTTACCAGAAGTACGGGTTTTCCCAGGA

AAATACTTTCCTTTAAAACAGTCCCTGATCACTCATCCTAAGAAAAGTCCAACTGAGTGTCTGAAGAAACAGGACTGCCCGCTGGCCCTGG
                                                                                  Kit-1 Small F
CTCAAAGATGCCCGAAGAGAGGAAGCTCCCCCCAGCCACTAGGAACTTGGCCTTTGGGGAATGACAAGTCCTGGGTTTGACATGGACGATG

TATGTGGAATGTGTAGGAAGAATTCCTCATTTCCACTGGGTTTTCCCCGCCATCCAACACAGCTCCCGCTGTAAATATGTAGGTTTCTGTG

TTTTGATTTCTGGAAAGCACTTATTGAAAGGTGAACCAGATAAGAAGCACCAAGGAGTGGTCTTTAATAAACACCAACCAGAACAAGGGCC
          Kit-1 target    PAM
CAGCCACCATCTGTGCGGCCGTTGGCTAGGGATGGTGTCGGGGCTGTCTGTCCATTCTTTTAACATTAACTTCCTTCACTTAAAGACCACT
                                                                                     Kit-1 Small R
GAGGACAAAGCAGGGGACAGGTCAAGAAGGGAGGCAAGGGTCAGCTCGGCCACACTCTAGAGAGCAGGACAGTTAGACTCCAGGCCACAGA

CAACTCAGCATATCCCTACTCCCAGCAGCCTCCCTGGCTGCATGGCGCACACGGGGCCTGAGGGGATAGACATAGGTGCTGGCCACAGGTC

CAGGTCAGGAGGCTTGGCCTGTGAGTGTGAATTTGGTGGTACCCTCCCAGCATGCCATGTTCtCTCAGGAGTTTCAGGATTCCGCTAAATG

CCAATTCTGCAGACCCAGGAGGGGATGCGAGGTGGTAATGGTGACAAGCCAAAAAAAAAAAAaaTAGGCTTTTAGGACCCTGGGCTCGATC

TCCAATCCCACAGCATCAAGTTCTAATGGTGTCGAACGAGGCATTAGGTAAGAAATGATTAGCATTTTCACACATCCATGTGTGTGTGTGT

GCACACATACATATGTACACGCATATGGCGACTGGGGCACTTCAGAAATGTTATTAGATAAAATATGCGTAATATTTAATTACCGCTTTAA
                                                 LTR
CCATTTGTAAGTATGCAGCTGAGTGGTGTACTAAGTCCTTTCGTGTGAAAAGAAAATTATATAAATTCTAGGTTTAAAAATATAAAAATTA

AAAAATAAACCCCAAAAGGCCACAGACCCAGGGCTAAGCCCTGCAGCCAAGACTAGCAGGCCATAAAGATAAAGGAGCACAGGAAACACTG
                                                                                     Kit-1 Large R
TTCAGGCAGGACTGACAAGCCATAAAAAAaGGAATGCAGGAACCAGCCTGAGTTAATGAGACTGATTCATGGGACGTCTGGCAGGAAGACA

TCTCCCCCCAGCTCACTCAGGGCCATATTTCAACTAGGTGTCCTCCAGCCCCTGATAAGCCCCTGACTTCTAGCACGTACTCTTTCTGCTT

TGTTCTCACTGCTATGTTTGAATGAGCCAATTGTATGTAACCACGCCAAAACCCCCTAGCCTTCTCTATATAACCCTCTGACTTTTGAGTT

TCGGGGCCGACACCTCTGTCTCCTGCGCGGGATACGTGTCGGCCCGGAGATCTCTGTAATAGGTCTCCGTAATAAACCTCGCCTTTGCTTA
                                                                                        ERV
TTACATCCAAAATGGTCTCTCTGTGTCTGGGGTCCGCGATTTCCCAAGACTTGAGTAAGGGTCTCTCTGCGTGGGATCTTTCATATTTATG

GGGGCTCGTCCGGGATCTGCGCGACTACCCCAGACTCCGAAGACCCCTTGGAGGTGAGTTGGATGTTTGTCTGAGTGTTTCTGTGTGAGCG

                                                 ERV(+5930bp)
ERV                                                                                     LTR
CAGTCAGTTAGGACAACTGGTGAGACCAAATAATACTTGATATCAAAATTCTAAGATTAGAATTATTTAATAGGAGAAGACAGGAATGAAA

AGAAAATTATATAAATTCTAGGTTTAAAAATATAAAAATTAAAAAATAAACCCCAAAAGGCCACAGACCCAGGGCTAAGCCCTGCAGCCAA

GACTAGCAGGCCATAAAGATAAAGGAGCACAGGAAACACTGTTCAGGCAGGACTGACAAGCCATAAAAAAaGGAATGCAGGAACCAGCCTG
                       Kit-2 Large F
AGTTAATGAGACTGATTCATGGGACGTCTGGCAGGAAGACATCTCCCCCCAGCTCACTCAGGGCCATATTTCAACTAGGTGTCCTCCAGCC

CCTGATAAGCCCCTGACTTCTAGCACGTACTCTTTCTGCTTTGTTCTCACTGCTATGTTTGAATGAGCCAATTGTATGTAACCACGCCAAA

ACCCCCTAGCCTTCTCTATATAACCCTCTGACTTTTGAGTTTCGGGGCCGACACCTCTGTCTCCTGCGCGGGATACGTGTCGGCCCGGAGA

TCTCTGTAATAGGTCTCCGTAATAAACCTCGCCTTTGCTTATTACATCCAAAATGGTCTCTCTGTGTCTGGGGTCCGCGATTTCCCAAGAC

TTGAGTAAGGGTCTCTCTGCGTGGGATCTTTCAGTGTGACTGGGCAGTCACCATCACTGTTCATTTCCTCACGTTTCCATCTTCCCCGCCC
                                                                Kit-2 Small F
AGCAGCTAGTACCTCTACACTCCTTTCTGCCCGGGCTCCCGAGCCCGCTTTCTATCTCTCTACAAACCGGGCCTTCTGGGTACCTTGAAGA

AGAGTCCGCATACGCTATTTGTTCTTGTGTGGCTGACATGTTTCCCTTGGAGTGTCACAGACATGCTGTACCATGTCAGAACCCCATCCCC
            Kit-2 target    PAM
ATTCAAGGCTAACGTTCCAGCGCTCGTTTGGGCCACACTTCTTTTTTTTCTACTCATCCTGACATTTGGATGGTTTCCCTGAGGCATTTT

CAACTGTGATCAACACTTTGGAGAGGCCTCTCTGACTTCCCAGCTTTGAAAGATGTTCCGCATTAATTACACTTGACTAAACTAGACGGGT
Kit-2 Small R
ATGCTAATTTCTTCATAATGGGTAAGTCAATACATTCTCCTCAGAAATATACTCTAAAAACTACTTTTAAGGAACCTGTCAAATAAGGAA

AATTGTACCTTCCCTTTTCCCACACAACTTGCAAACAGGAAATTTTTCCTTCTGTTTCTACTGTCAAGATGTCATCTTACGGAGGCGGGGA

CGAAAGCAGTCATGTTTCCCACAAGAAACTGTACCTGAGGCAGTTGCTGGAGGAAAGGGCAGAGGCAGTTGTTAAACTGTTCTGCCACAGA

CTTCTACTCCAGGGCGACCGTACACTGCAGCCCTGGCTAACAGGCCCCGGGCAAATCCCGCAGAGAACCATTTGGTGAGCCCATCTCTCCC

GTCTTCCTGTTACCTTGCCAAAGGGGAGAATCTGTTTTGTAGGTGCTAGAGATTTCCAGGAAGACAGCAAGTCTGTGTAGAACTTGCAGAA
                                                                                    Kit-2 Large R
CTTAAGGGGGCTCCCTGAGGAACTCAAAGCACCCTTCTTGGTGGGAAGCCTTACCAGAAGGAAGGAAAATAGTCTCTGGTCAAGGGCAGAG
```

Supplementary Figure 14. Two gRNAs designed to target both neighbouring sequences outside of the endogenous retrovirus (ERV) element within the first intron of the *Kit* gene. The two gRNA-binding sequences (blue) and the PAM sequences (green) are shown at each outer side of the two LTR sequences (yellow) and the ERV (green). The ssODN:*Kit*^{H} sequences consisting of two 60-bp fragments at each of the outer sides of the Cas9-cutting edges are underlined. The primer sets (small and large) used for the PCR analysis are shown in boxes.

Supplementary Table 1. Germline transmission in the offspring of CRISPR/Cas modified rats

Founder (G0) No.	CRISPR-mediated mutation	G1 offspring		G1 mutants	
		males	females	males	females
#1	+1	15	8	3	0
#2	+3	17	25	7	10
	+2			2	2
	-3			3	2
	-5			5	11
#3	-1	18	15	5	4

Supplementary Table 2. Potential off-target sites for CRISPR/Cas targeting on the rat genome

Name	Sequence	Hit score*	No. of Mismatch	Coordinate (rn5)	Strand	Indel Mutation frequency (Mutant/Total)
gRNA:*Tyr*^{c}	TTTCCAGGATTATGTAATAGTGG					
Tyr^{c}-OT1	CTCCCAGGGTTATGTAATAGAAG	1.6	4	chr19:32965297-32965319	+	0/7
Tyr^{c}-OT2	TATCCAGGCATATGTAATAGCAG	1.5	4	chr10:32914681-32914703	-	0/7
Tyr^{c}-OT3	ATTCCAGGAGTATGTAATACTAG	1.4	4	chr19:24325208-24325230	+	0/7
Tyr^{c}-OT4	TTTCCAGAACTATGTAATACTGG	1.1	3	chr11:34484150-34484172	+	0/7
Tyr^{c}-OT5	TTTCCATGTGTATGTAATAGAAG	0.9	4	chr4:22739080-22739102	+	0/7
Tyr^{c}-OT6	TTACTATTATTATGTAATAGTAG	0.9	5	chr10:78685738-78685760	-	0/7
Tyr^{c}-OT7	TTGCTATGACTATGTAATAGGAG	0.9	5	chr13:118286168-118286190	-	0/7
gRNA:*Asip*^{a}	ATGACAGGAGTCTAACTCACTGG					
Asip^{a}-OT1	CTGACAGGAGTCTAACTCAGGAG	10.4	3	chr17:60056463-60053485	+	0/11
Asip^{a}-OT2	ATCCCAGGACTCTAACTCACTAG	2.4	4	chr13:90729314-90729336	+	0/11
Asip^{a}-OT3	ATTTCATGAGTCTAACTCACTAG	1.6	4	chr7:91031972-91031994	+	0/11
Asip^{a}-OT4	CTCACAGAATTCTAACTCACTGG	1.3	4	chr4:160198608-160198630	+	0/11
Asip^{a}-OT5	ATGTCAGGAATCTAACTCAAAGG	1.3	3	chr6:3123384-3123406	+	0/11
Asip^{a}-OT6	CAGAAATGAGTCTAACTCACAGG	0.9	4	chr2:126359547-126359569	+	0/11
Asip^{a}-OT7	CAGAAATGAGTCTAACTCACAGG	0.9	4	chr5:117771022-117771044	-	0/11
Asip^{a}-OT8	ATGCTATCAGTCTAACTCACAAG	0.9	5	chr6:56158010-56158032	+	0/11
Asip^{a}-OT9	AGGAGAGGAGTCTAGCTCACCAG	0.8	4	chr16:80891004-80891026	-	0/11
Asip^{a}-OT10	CATACTGGAGTCTAACTCACAAG	0.8	5	chrX:123861706-123861728	+	0/11
Asip^{a}-OT11	CTCACAGGTCTCTAACTCACTGG	0.8	4	chr9:95170875-95170897	+	0/11
gRNA:*Kit*^{h}*-1*	CATCTGTGCGGCCGTTGGCTAGG					
Kit^{h}-1-OT1	CAGCTGTGCTGCCGCTGGCTGAG	0.7	4	chr6:76541217-76541239	+	0/11
Kit^{h}-1-OT2	GAGCTGTGCAGGCGTTGGCTGAG	0.7	5	chr7:114146101-114146123	+	0/11
Kit^{h}-1-OT3	CAGCTGTGTGGCAGTTGGCTGAG	0.7	4	chr11:73983869-73983891	-	0/11
Kit^{h}-1-OT4	AATCTGTACTGCCGTTGGCAGAG	0.7	5	chr5:120208583-120208605	-	0/11
gRNA:*Kit*^{h}*-2*	CTAACGTTCCAGCGCTCGTTTGG					
Kit^{h}-2-OT1	CTAATGTTCTAGGGCTCGTTAAG	1	4	chr15:63469421-63469443	-	0/11
Kit^{h}-2-OT2	CTTCTGTTCCAGCGTTCGTTCAG	0.4	5	chr9:99298933-99298955	-	0/11
Kit^{h}-2-OT3	CTCCGGTTCCAGCGCTCCTTCAG	0.3	5	chr9:15473616-15473638	-	0/11
Kit^{h}-2-OT4	CTCCGGTTCCAGCGCTCCTTCAG	0.3	5	chr9:15231390-15231412	-	0/11
Kit^{h}-2-OT5	CTAACTTTCCAAGGCTCGTTAAG	0.3	4	chr1:244875880-244875902	-	0/11

*Off-target hit scores are calculated by CRISPR Design Tool (http://crispr.mit.edu/).

Supplementary Table 3. Oligonucleotides used for off-target analysis

Name	Direction	Sequence (5' to 3')
Tyr^{c}-OT1	F	TTGAATCCTGTGATACAGTTCAGAC
	R	CATATGCCGAGGTCACAACA
Tyr^{c}-OT2	F	TGGCCATACTCCAAATCTTCTT
	R	CCCTGATCCTGTAGTTTTGTGA
Tyr^{c}-OT3	F	GGACACCTGCAGTAAGGACA
	R	TGAGATTGAGATCATTTTGGTCA
Tyr^{c}-OT4	F	GAGTGAGCAATGGCGGATAC
	R	CACAGTTGCTCACACGCTTT
Tyr^{c}-OT5	F	TGTGGTATCAGCTGGAGCAT
	R	CAGAGCAGATGGCACTCCTA
Tyr^{c}-OT6	F	AGGCGAGGATGTATTGGTTG
	R	CATGCATGTGAAGCAGGACT
Tyr^{c}-OT7	F	TCAACCACAGGTTTGGGAAT
	R	CTCTTGTTCATGGCCTCTCC
Asip^{a}-OT1	F	CATGAGGTAAACCACTTTGCTC
	R	TCAAGAAGTCCCCTCAGTCA
Asip^{a}-OT2	F	ATGAGGGTGGGGACTGCTAT
	R	CTGGATTTTCCCAGCTGATG
Asip^{a}-OT3	F	AAACTGGCTGGACCAATCTG
	R	AAGCCAAGCCTTGTATGAGC
Asip^{a}-OT4	F	TGCTCCAGTGTTTGCTGAAG
	R	TTTGTGACCATGTCTGCTCTT
Asip^{a}-OT5	F	GCAGTGAGACCTAAGCAGACC
	R	GGGGGCAGTAGGAAGAGATT
Asip^{a}-OT6	F	CAAGGTCTTTAGGGGCTGTG
	R	TGACACTAGAACATCAAACCATTTC
Asip^{a}-OT7	F	CATTTGCTGTTCCACAGCTT
	R	CCACCATCAACAATCACCAG
Asip^{a}-OT8	F	CGCACAACTCAAACTTTGGATA
	R	TCCCAGTGGAGAGGAATGTC
Asip^{a}-OT9	F	GAAGCCTCTAACAGCAACACG
	R	ACCACTGCCAGGCACTAACT
Asip^{a}-OT10	F	CTGAGCTGCCCTTTCTTAGC
	R	GAATTTGCACCATTTCAAAAA
Asip^{a}-OT11	F	AGCAGAAGCATTAACCCAGA
	R	CTTGCCTAGGAAGCGCAAGG
Kit^{h}-1-OT1	F	TTTGCCAAGTATTGCACCAA
	R	GGCCATCACCTCTGCACTTA
Kit^{h}-1-OT2	F	CGCCACCAAAACCATTTAAG
	R	AACCCTACAGTTTGCGATGC
Kit^{h}-1-OT3	F	CACCAAGGTCCCTCAGACAG
	R	AGTATAACCCTGCCGCCTTC
Kit^{h}-1-OT4	F	GGCAAGGCATATTGAGAAGG
	R	TATCTCCCTCTCTGCGCCTA
Kit^{h}-2-OT1	F	ACACCAGCTTCCAAAACCAG
	R	CAACAGTCCCAAAGTCTGAGC
Kit^{h}-2-OT2	F	TCCTCATTGGACCTCTGCTC
	R	TGTGACAAACCCACAGCATT
Kit^{h}-2-OT3	F	TGATGTCAAAGCCTCCCTTT
	R	GCAGAGGAAAGGACTCTGGA
Kit^{h}-2-OT4	F	GGTCAGAGGAACTGCACCTT
	R	GACCCTCTGACTGGGCTCTC
Kit^{h}-2-OT5	F	AGGCCAGCTACCAGAACTCA
	R	ATCCCAAAGTCCAAGCTTCC
